# *In vivo* melanin 3D quantification and z-epidermal distribution by multiphoton FLIM, phasor and Pseudo-FLIM analyses

**DOI:** 10.1038/s41598-021-03114-0

**Published:** 2022-01-31

**Authors:** Ana-Maria Pena, Etienne Decencière, Sébastien Brizion, Peggy Sextius, Serge Koudoro, Thérèse Baldeweck, Emmanuelle Tancrède-Bohin

**Affiliations:** 1 L’Oréal Research and Innovation, 1 Avenue Eugène Schueller, BP22, 93601 Aulnay-sous-Bois, France; 2 MINES ParisTech – PSL Research University, Fontainebleau, France; 3 L’Oréal Research and Innovation, Campus Charles Zviak RIO, 9 rue Pierre Dreyfus, Clichy, France; 4 Service de Dermatologie, Hôpital Saint-Louis, Paris, France

**Keywords:** Multiphoton microscopy, Three-dimensional imaging

## Abstract

Characterizing melanins *in situ* and determining their 3D z-epidermal distribution is paramount for understanding physiological/pathological processes of melanin neosynthesis, transfer, degradation or modulation with external UV exposure or cosmetic/pharmaceutical products. Multiphoton fluorescence intensity- and lifetime-based approaches have been shown to afford melanin detection, but how can one quantify melanin *in vivo* in 3D from multiphoton fluorescence lifetime (FLIM) data, especially since FLIM imaging requires long image acquisition times not compatible with 3D imaging in a clinical setup? We propose an approach combining (i) multiphoton FLIM, (ii) fast image acquisition times, and (iii) a melanin detection method called Pseudo-FLIM, based on slope analysis of autofluorescence intensity decays from temporally binned data. We compare Pseudo-FLIM to FLIM bi-exponential and phasor analyses of synthetic melanin, melanocytes/keratinocytes coculture and *in vivo* human skin. Using parameters of global 3D epidermal melanin density and z-epidermal distribution profile, we provide first insights into the *in vivo* knowledge of 3D melanin modulations with constitutive pigmentation versus ethnicity, with seasonality over 1 year and with topical application of retinoic acid or retinol on human skin. Applications of Pseudo-FLIM based melanin detection encompass physiological, pathological, or environmental factors-induced pigmentation modulations up to whitening, anti-photoaging, or photoprotection products evaluation.

## Introduction

Characterizing melanins in their native environment and determining their 3D distribution in epidermis is paramount for understanding physiological processes of melanin neosynthesis, transfer and degradation and their modulation with external factors such as UV light exposure, topical products application, or skin diseases.

Human skin melanins, are a mixture of eumelanins and pheomelanins^1–5^ produced in melanosomes organelles within the melanocytes^6^. Stage IV melanized melanosomes are transferred to keratinocytes and their number, size and distribution within the epidermis contribute along with other chromophores mainly hemoglobin to human skin color^1^.

Currently, the gold standard method for melanin quantification in skin is high-performance liquid chromatography (HPLC) chemical analysis of melanin degradation products^7,8^. Although very specific, it requires *ex vivo* samples degradation and provides no information on melanin’s epidermal distribution. Fontana-Masson staining of transverse skin sections allows insights in 2D melanin distribution and content (e.g.^9,10^) but has the drawback of non-specific *stratum corneum* (SC) staining, while Warthin-Starry stain may provide a more sensitive and specific melanin detection^11^. Transmission electron microscopy enables melanosomes pattern analysis within epidermal cells and its variations according to skin phenotype (e.g.^10^).

Assessing melanin content and distribution *in vivo*, in 3D, is still a challenging topic. Over the years, the possibility to provide a non-invasive melanin detection based on its optical properties, broad-band UV and visible absorption^12,13^, fluorescence emission spectrum and lifetime^14,15^, was investigated using different techniques. In confocal reflectance microscopy^16^, melanin contrast arises from refractive index changes between melanin pigment and other constituents^17–19^, but cellular membranes and corneocytes also exhibit similar reflection signals, making specific detection and quantification impossible. Studies using spontaneous Raman spectroscopy found eumelanin and pheomelanin to have specific peaks and the 2000 cm^−1^ peak of pheomelanin located within the “silent region” of the Raman spectrum seems to offer a straightforward route to specific non-invasive 3D pheomelanin detection in skin samples by CARS (Coherent anti-Stokes Raman Scattering) imaging^20^. The Raman bands 550–1200 and 1650–2300 cm^−1^ were also shown to be significant to predict the ratio of eumelanin subunits^21^. Recently, it was proposed that a combined analysis of specific Raman bands along with the NIR one-photon excited skin autofluorescence^22,23^ could be used to estimate an xz depth-profile of melanin fraction *in vivo*^24^ but this method lacks spatial localization of melanin within the cells and cannot image the entire epidermis. Pump-probe imaging has also been proven of value in analyzing melanin within 2D thin skin sections of pigmented lesions, namely for its eumelanin/pheomelanin discrimination^25–27^. As the dominant absorber within epidermis, melanin also provides a strong third harmonic generation (THG) signal in the basal layers and, using an *in vivo* calibration method, a melanin mass density can be estimated from THG images^28^. However, it is not clear how melanin THG signal is discriminated from the intense THG signals of ordered lipid assemblies within SC^29^.

Melanin imaging based on its endogenous fluorescence was evidenced in 1979 by conventional one-photon excited fluorescence microscopy on human skin sections^30^ and 20 years later *in vivo* on forearm skin using two-photon excitation^31^. Multiphoton imaging enables *in vivo* human epidermis and superficial dermis characterization up to a depth of ~ 200 µm^32,33^. In addition to detecting intrinsic fluorescence from cellular and extracellular matrix constituents (e.g. keratin, NAD(P)H, FAD, melanin, and elastin) and second harmonic generation signals from fibrillar collagens^34–36^, multiphoton microscopy can be leveraged using fluorescence lifetime imaging (FLIM) to provide functional information on skin constituents^37^. Fluorescence lifetime is independent of the fluorophore concentration, but depends on the local microenvironment of the molecule, on variables such as pH, binding status, and molecular conformational changes. The skin’s autofluorescence lifetime (see^38^ and included references) spans from hundreds of picoseconds (e.g. melanin, free NAD(P)H, bound FAD) to nanoseconds (e.g. bound NAD(P)H, free FAD, keratin). Multiphoton FLIM imaging of melanin samples such as synthetic, Dopa or *Sepia* melanins, skin and eye melanocytes, human hair and hair bulb, and human skin (e.g.^38–43^) indicate a specific bi-exponential decay behavior with a predominantly (> 90% relative contribution) short-fluorescence lifetime component around ~ 100–200 ps and a mixed species phasor plot with short phase lifetime distribution. However image acquisition time needed for acquiring correct fluorescence decays for bi-exponential or phasor^42–44^ analysis is not compatible with 3D skin imaging in a clinical setup and in practice is limited to 2D imaging at selected epidermal depths.

Using *in vivo* 3D multiphoton imaging at 760 nm excitation, we were the firsts to propose an intensity-based melanin detection method. We compared it to histology Fontana-Masson and verified the 3D melanin global density modulation on human forearm skin upon topical application of corticosteroids *in vivo*^45^ and *in vitro* on reconstructed pigmented epidermis^46^. An intensity-based melanin detection upon 880 nm excitation was later used to characterize different skin color pigmentation phototypes^47^. This intensity-based approach works in the basal epidermal layers where melanin is highly concentrated and exhibits 2PEF signal intensities stronger than other endogenous fluorophores, but not in SC containing keratins with high fluorescence intensities. Moreover, pixels with low melanin concentration, low fluorescence intensity are not taken into account.

In order to detect melanin from multiphoton FLIM-like data compatible with 3D *in vivo* acquisitions on human subjects, we propose to use an approach combining (i) multiphoton FLIM, (ii) fast image acquisition times, and iii) a melanin detection method, that we call Pseudo-FLIM, which is based on slope analysis of the 2PEF intensity decay from temporally binned data^48,49^. In this paper, we validate our approach on synthetic melanin, melanocytes and keratinocytes coculture and *in vivo* human skin and compare it to FLIM bi-exponential and phasor analyses. Using parameters of melanin 3D global density and z-epidermal distribution, we assessed *in vivo* melanin modulations under different conditions: constitutive and acquired pigmentation, aging, natural UV exposure or application of topical retinoids known to having an effect on pigmentation.

## Materials and methods

### *In vitro* normal human melanocytes/keratinocytes (NHMK) coculture and synthetic melanin samples

Synthetic melanin (prepared by oxidation of tyrosine with hydrogen peroxide, Sigma-Aldrich M0418) dispersed in water was placed between a glass slide (VWR) and a glass coverslip (Marienfield 24 × 50 mm^2^) using an adhesive silicon isolator (Grace Bio-Labs, JTR8R-0.5).

Normal human melanocytes/keratinocytes (NHMK) (see supplementary materials and methods) were grown in 96 well plates for 7 days.

### *In vivo* human skin: clinical trials

All clinical studies were conducted in Paris, France (all volunteers gave written, informed consent and experimental protocols were approved by the Saint Louis Hospital ethics committee (EC), complying with the Declaration of Helsinki).

*Constitutive pigmentation* study (February–March 2010, EC reference 2010/01; original data). This study involved 37 female volunteers (18–55 y) with skin color determined by Individual Typology Angle (ITA): > 55° (ITA group I, “very light skin”, n = 4 European origin (Eu.O) and n = 1 Asiatic origin (As.O));]41°, 55°] (ITA group II, “light skin”, n = 5 Eu.O and n = 5 As.O);]28°, 41°] (ITA group III, “intermediate skin”, n = 5 Eu.O and n = 5 As.O);]10°, 28°] (ITA group IV, “tanned skin”, n = 4 Eu.O, n = 2 As.O and n = 1 African origin (Af.O));]30°, 10°] (ITA group V, “brown skin”, n = 5 Af.O). Multiphoton and colorimetry (Datacolor, Montreuil, France) measurements were performed on ventral forearm. This study allowed to determine the changes in melanin global density and z-epidermal distribution with both skin color and ethnicity.

*Photo-aging* study (January–February 2009, EC reference 2008/62; parameters of melanin characterization are original data, other data from this study having already been published^50^,) This study involved 15 young (18–25 y) and 15 aged (70–75 y) female volunteers with Fitzpatrick phototypes I to IV. Imaging was performed on ventral and dorsal (mostly unexposed vs. exposed) forearms. Melanin modulations in this context revealed chronic cumulative sun exposure at two different ages.

*Retinoids under occlusion* study (March–July 2012, EC reference 2012/05; published^51^). This study involved 20 female volunteers (50–65 y) with skin color ITA between 10° and 41° (ITA group III/IV). Two products, Retinol 0.3% (RO, Retinol 0.3®, L’Oréal product) and all-trans Retinoic acid 0.025% (RA, Retacnyl®, Galderma) were applied under occlusion on dorsal forearm side for 12 days. A third untreated, occluded area was used as control. Imaging was performed at days D0, D12 (end of occlusion period), D18 and D32. This study aimed at studying effects of reference anti-aging products on melanin among other parameters in a short-term *in vivo* model.

*Retinoids 1-year* study (February 2011–April 2012, EC reference 2010/58; published^52^) This study involved 30 female volunteers (50–65 y) with skin color ITA between 10° and 41° (ITA group III/IV). They applied Retinol 0.3% (RO, Retinol 0.3% cream, L’Oréal group) (n = 15) or all-trans-Retinoic acid 0.025% (RA, Retinoic acid 0.025% cream, Galderma) (n = 15) on one dorsal forearm versus a control product (white paraffin containing excipient, Bayer) on the other forearm for 1 year. Imaging was performed at months M00 (March), M03 (June), M06 (September), M12 (March + 1 yr). This study aimed at studying effects of reference anti-aging products on melanin among other parameters in “real life” applications; acquisitions on the control conditions allowed to study melanin modulation with seasonality.

### *In vitro* 2D multiphoton FLIM imaging

Multiphoton FLIM imaging (2D 2PEF FLIM) of synthetic melanin and NHMK coculture was performed with a commercial microscope (Leica TCS SP8 MP FLIM, Leica, Germany) integrating a time-correlated single photon counting (TCSPC) PicoHarp 300 module (Picoquant, Germany) and an 80 MHz IR fs pulsed laser (Newport Chameleon Ultra II, 680–1080 nm). Images were acquired upon 760 nm excitation and detection in the 410–650 nm range using a 40×/1.1 NA water immersion objective. Images of 205 × 205 µm^2^ (512 × 512 pixels × 3128 time channels; 4 ps/time channel, 0–12.5 ns time range, position of the peak of the intensity decay at 1.33 ns) were acquired with 1.2 µs pixel dwell time, 50 frame accumulation number at 2 mW for melanin solution, and respectively 200 for NHMK coculture at 5 mW.

### *In vivo* 2D multiphoton FLIM imaging

Imaging at different skin depths was performed using DermaInspect™ (JenLab GmbH, Jena, Germany) medical device integrating TCSPC detectors, a FLIM module (SPC-830, Becker & Hickl, Berlin, Germany) and an 80 MHz IR fs pulsed laser (MaiTai Spectra-Physics, Mountain View, CA, USA). Images of 130 × 130 µm^2^ (511 × 511 pixels; 51 µs pixel dwell time) were acquired upon 760 nm excitation and detection in the 390 –650 nm range using a 40×/1.3 NA oil immersion objective (exponentially increased excitation power from 12 mW at the skin surface up to 47 mW at depths exceeding 75 µm). The signals from 4 pixels were combined during acquisition with SPCM software to give 127 × 127 pixels × 256 time channels images (48.8 ps/time channel, 0–12.5 ns time range, maximum intensity decay at 1.33 ns; 13.4 s/image; 104 µs/binned pixel).

### *In vivo* 3D multiphoton imaging: 2PEF-FLIM (4 time channels)/SHG z-stacks

Multiphoton imaging (DermaInspect™, Jenlab, Germany) was performed as previously described^45^. For each experimental condition, we acquired two adjacent 3D z-stacks (70 images; 2.346 µm z-step). Image characteristics: 511 × 511 pixels (0.255 µm/pixel) × 4 time channels (2.08 ns/time channel), 0–8.33 ns time range (when acquiring 4 time channel images, the time range needed to be decreased from 12.5 ns to a software predefined value of 8.33 ns in order to achieve the ~ 2 ns temporal binning for the Pseudo-FLIM analysis), maximum intensity decay at 1.33 ns; 28 µs pixel dwell time; 7.4 s/image; 9.4 min /3D z-stack.

### FLIM bi-exponential analysis

**Figure 1:**
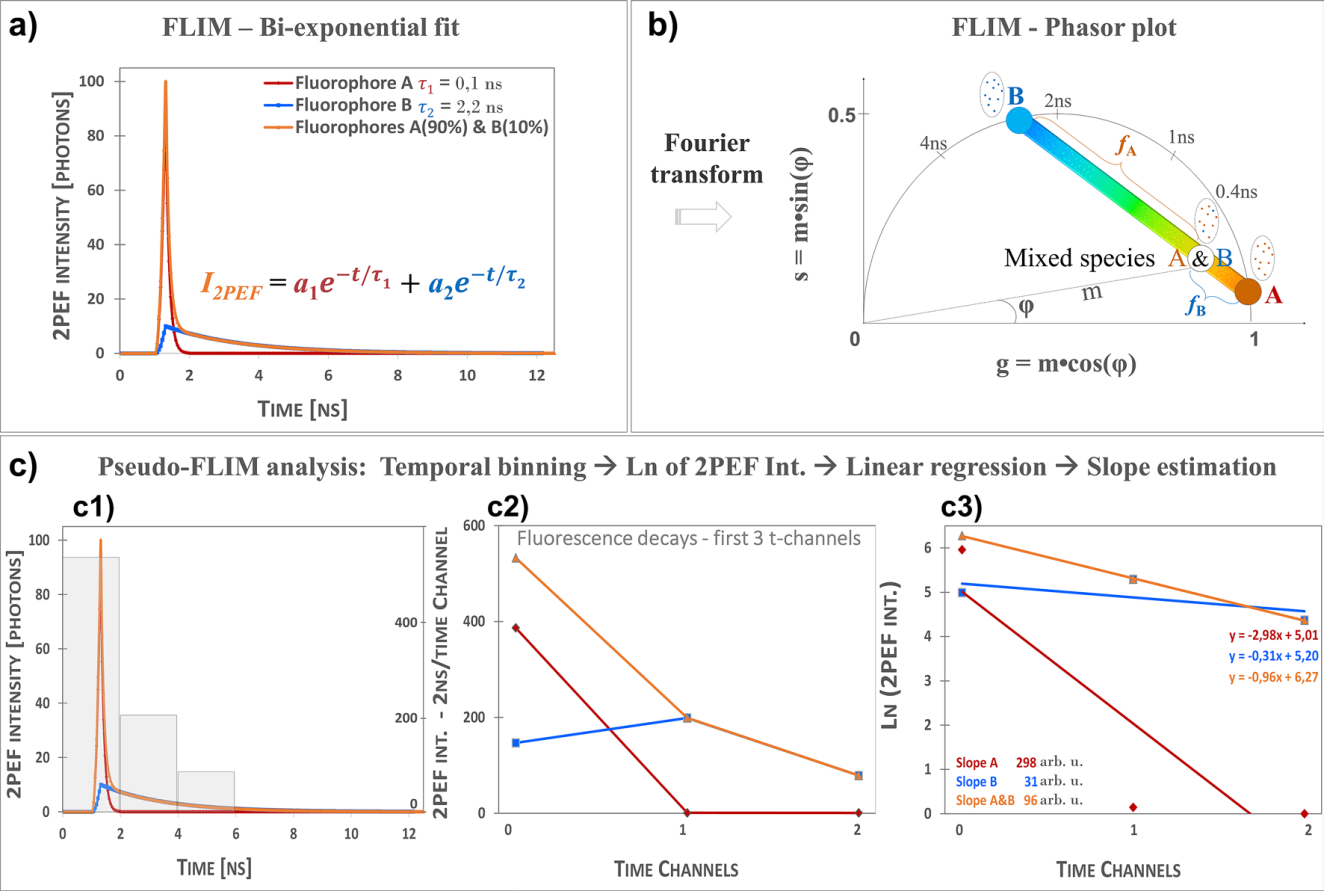
Principle of FLIM bi-exponential, Phasor and Pseudo-FLIM analyses. (**a**) Simulated mono-exponential (fluorophores A and B) and bi-exponential (mixed species of fluorophores A and B with respectively 90% and 10% relative contribution) two-photon excited fluorescence intensity decays (12.5 ns time range; 80 MHz); this shape of A&B bi-exponential decay can be measured in melanin containing samples. In FLIM bi-exponential analysis, the 2PEF intensity decay is adjusted with the function in (**a**) to compute the values of short- ${\tau }_{1}$ and long-${\tau }_{2}$ fluorescence lifetimes and their relative contributions ${a}_{1}\left[\%\right]$ and ${a}_{2}\left[\%\right]$. Images of FLIM bi-exponential fit and combination parameters such as amplitude- and intensity-weighted lifetimes are used for data analyses. (**b**) FLIM phasor analysis transforms a decay into a phasor with polar coordinates *g*, *s*, corresponding to the real and complex components of the Fourier transform, that can also be expressed as a function of *m* modulation and *φ* phase angle. Mono-exponential decays such as A and B will have their phasors on the semi-circle, whereas mixed species will have a phasor along a line connecting the two distinct lifetime phasors of A and B. The relative fractions ${f}_{A}$ and ${f}_{B}$ can be computed from the distances of A&B mixed species phasor to B and respectively A phasors. Images of *g* and *s* as well as combination parameters such as the apparent phase and modulation lifetimes and their relative fractions are used for data analyses. (**c**) Pseudo-FLIM analysis firstly involves a temporal binning of the 2PEF decay into a reduced number of time channels with 2 ns integration time per channel (see **c1**, gray bars indicate the photon intensity of the mixed A&B species within the first 3-time channels with 2 ns binning). The 2PEF intensity of the binned first 3-time channels for simulated A, B and mixed A&B species is given in (**c2**). After a natural logarithm transformation (**c3**), a linear regression of the first 3-time channels is performed to calculate the slope of the decay which is multiplied by a − 100 factor to create the Pseudo-FLIM slope parameter. The faster the decay, the higher the slope. Pseudo-FLIM image of the slope parameter is further processed for melanin detection by applying a threshold to keep the pixels with high slope values.

FLIM parameters were computed by fitting the fluorescence intensity decay with a bi-exponential function^38–41^ ${{{I}_{2PEF}(t)=a}_{1}e}^{- \frac{t}{{\tau }_{1}}} + {a}_{2}{e}^{- \frac{t}{{\tau }_{2}}}$ to extract the short and long fluorescence lifetimes ${\tau }_{1}$ and ${\tau }_{2}$, their relative amplitudes ${a}_{1}\left[\%\right]=\frac{{a}_{1}}{{a}_{1}+{a}_{2}}$ and ${a}_{2}\left[\%\right]=\frac{{a}_{2}}{{a}_{1}+{a}_{2}}$ and compute amplitude-weighted ${\tau }_{Av Amp}=\frac{{a}_{1}{\tau }_{1}+{a}_{2}{\tau }_{2}}{{a}_{1}+{a}_{2}}$ or intensity-weighted ${\tau }_{Av Int}=\frac{{a}_{1}{\tau }_{1}^2+{a}_{2}{\tau }_{2}^2}{{a}_{1}{\tau }_{1}+{a}_{2}{\tau }_{2}}$ average lifetimes. Simple fluorescent molecules have single exponential decays (e.g. simulated decays Fig. 1a), but in skin, the shape of this decay varies depending on the local microenvironment (e.g. pH, binding status, molecular conformational changes) of the fluorophores and on the mixed ratio of fluorescent signals arising from free/bound NAD(P)H, free/bound FAD, keratin, melanin, elastin, etc. Pixels with mostly melanin, as a main contributor to the detected signal, will exhibit a decay similar to the simulated A&B mixed species decay (Fig. 1a).

FLIM bi-exponential analysis of *in vitro* (512 × 512 pixels × 3128 time channels) and *in vivo* (128 × 128 pixels × 256 time channels) data is described in the supplementary materials and methods.

### FLIM phasor analysis

By applying a Fourier transform to every pixel, phasor method^44,53,54^ transforms the 2PEF intensity decay into a phasor with coordinates g and s within the phasor plot (Fig. 1b, see details in Supplementary materials and methods).

In human skin, the 2PEF signal decays as a sum of multiple exponentials and is a mixture of two or more lifetime components originating either from two different molecules (e.g. free NAD(P)H and bound NAD(P)H), a single molecule (e.g. melanin, elastin) or a mixture of molecules (e.g. melanin and free-/bound-NAD(P)H, keratin and free-/bound-NAD(P)H, etc.). Consequently, the phasor plot of human skin lies within the universal semicircle and reflects the mixed molecular species contributions. By observing the pixels clustering in the phasor plot and by mapping the clusters to their corresponding *i*,*j* pixels within the FLIM image, skin layers or cells with similar phasors can be identified. One can also use the morphological/structural tissue information to attribute the phasor cluster to a specific skin constituent (e.g. elastin within the elastic fibers).

Phasor analysis was performed with SPCImage (v 8.1, Becker & Hickl, Berlin, Germany) and home-written macro with Fiji/ImageJ (W. Rasband, NIH, USA). After bi-exponential analysis, g and s parameters were calculated using the same fixed shift parameter for all the images and phasor plot images were color coded for ${\tau }_{1}$ or ${\tau }_{2}$ fluorescence lifetimes. Exported g and s images were further analyzed with the Fiji macro to calculate the phase ${\tau }_{\varphi }$ and modulation ${\tau }_{m}$ lifetimes. All these images were further processed for melanin quantification with Fiji. Phasor plot color coded images were modified with Fiji to change the black background color to white.

### Pseudo-FLIM analysis for 2D and 3D melanin detection

FLIM images with different number of time-channels and temporal binning can be analyzed by Pseudo-FLIM for melanin detection^48,49^ (see Fig. 1c). The main idea is to bin the fluorescence photons in a reduced number of time channels either at the acquisition or afterwards during processing and perform a linear regression to estimate the slope of the decay. FLIM 2PEF intensity decays with a peak position (maximum of 2PEF intensity decay) at 1,33 ns are temporally binned into channels with 2 ns per time channel (Fig. 1c1). The first 3 time-channels are used for further analysis. Figure 1c2 shows the simulated 2PEF intensity decays with 2 ns temporally binned channels of fluorophores A, B and mixed species A&B. Mono-exponential short-fluorescence lifetime decays (e.g. A, 0.1 ns) will have a higher 2PEF intensity in the first binned time-channel compared to long-fluorescence lifetime decays (e.g. B, 2.2 ns). The photons in this first binned time-channel are an integral of noise photons (detected before the rising edge) and fluorescence photons. The higher the photon intensity within this 2 ns time window, the higher the slope of the decay (see Fig. 1c2 and c3). The decays are transformed in ln(2PEF Intensity) and a linear regression is performed to extract the slope of the decay (Fig. 1c3). In this example, fluorophore A (${\tau }_{1}$=0.1 ns) has a higher slope compared to fluorophore B (${\tau }_{2}$=2.2 ns). Mixed species A&B decay with 90% contribution of A has also a high slope compared to B, but smaller than A. In tissues, the difference between different mixed species decays will come from differences in both fluorescence lifetimes and in their relative contributions. Fast decays with high relative contributions will have high Pseudo-FLIM slopes.

Figure 1 illustrates Pseudo-FLIM analysis at the pixel level. When analyzing images, we first calculate the temporal binning and apply a Gaussian blur with a radius $r=\sqrt{\frac{Melanin filter area/d{x}^{2}}{\pi }}$ where *dx* is the pixel size in µm and melanin filter area was fixed to a value of 1 µm^2^ proximal to the melanosome area. As Pseudo-FLIM melanin analysis requires only few photons per pixel (see “Results”section), it results in faster image acquisition compatible with 3D imaging on humans. After temporal binning and Gaussian blurring, some pixels in the 3D z-stack of 2PEF FLIM (4 time channels) images will have zero values (images acquired above the skin surface, sometimes pixels within less or non-fluorescent regions such as nuclei and cellular membranes). Before calculating the natural logarithm, we add 1 photon to all the pixels of the temporally binned images. A Pseudo-FLIM slope parametric 8 bits image is created by multiplying the slope of the decay (results of the linear regression fit; mostly negative values) by a factor of −100 to have positive values and the pixels with values above 255 are set to 255 and below 0 to 0. We express the Pseudo-FLIM slope parameter in arbitrary units, but one could also divide its value by the time channel duration and express it in 1/ns units (as it is done for single exponential decays: the decay time $\tau$ can be calculated from the slope of a plot of *log I(t)* versus *t*, or from the time at which the intensity decreases to 1/*e* of the intensity at *t* = 0 and the slope $=-1/\tau$)^55^.

Pseudo-FLIM slope parametric images are further processed for melanin mask calculation by applying a threshold to keep the high slope values pixels (above 70) mainly corresponding to melanin (see “Results”). We also apply an open area filter to remove the isolated regions of interest with an area smaller than the approximated 1 µm^2^ melanosomal area. The influence of the temporal binning and of the maximum intensity peak position on Pseudo-FLIM results is described in supplementary materials and methods (Fig. S1).

We implemented Pseudo-FLIM analysis with both Python in Multiphoton Skin Tools Suite (MPSTS) software^50^ and using a home-written macro with Fiji/ImageJ.

### *In vivo* 3D automatic skin layers segmentation and constituents quantification

**Figure 2:**
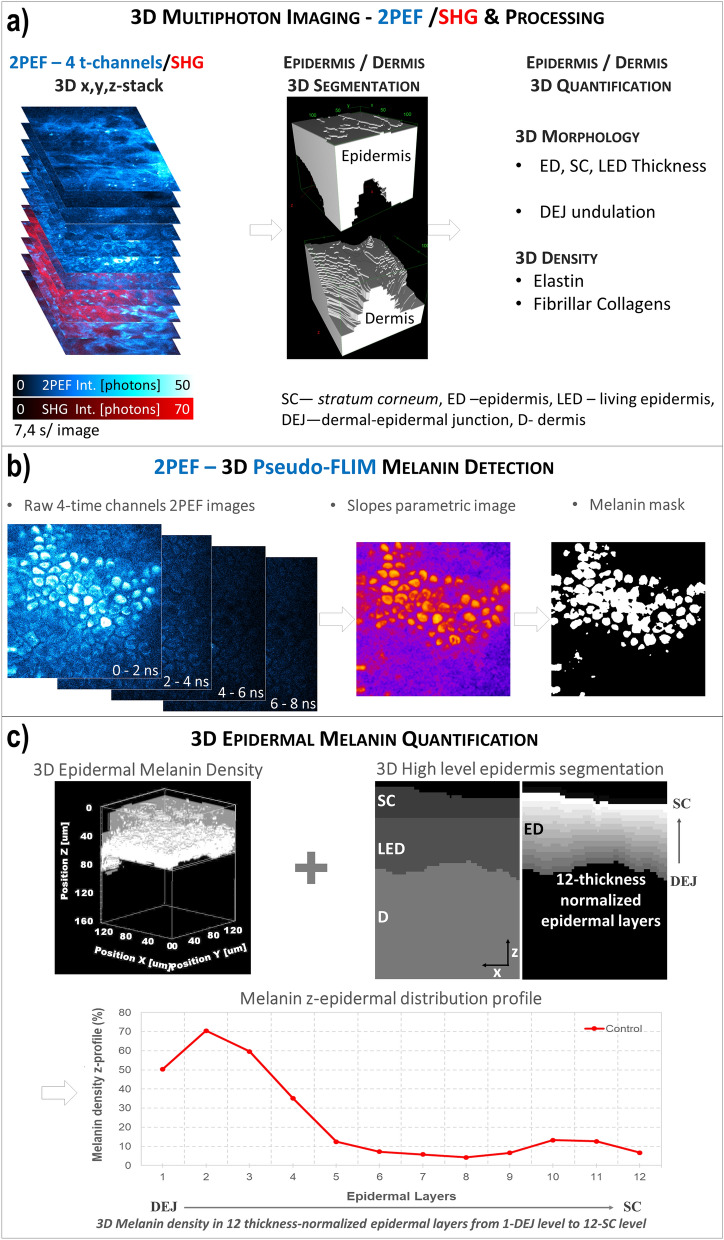
Global analysis process for *in vivo* 3D multiphoton images allowing 3D skin automatic layers segmentation and constituents quantification. (**a**) The first step of global 3D analysis of z-stacks of combined 2PEF-FLIM (4 time channels)/SHG images briefly consists in identifying the epidermal and dermal layers (3D automatic segmentation) and quantifying their morphology (thickness, DEJ 3D-shape) and the 3D dermal density of elastin and fibrillar collagens. (**b**) The z-stack of 2PEF-FLIM (4 time channels) images is further processed for melanin detection using Pseudo-FLIM method. The first 3-time channels are used for slope parametric image calculation and a melanin mask is obtained by applying a threshold to keep the high slopes values (above 70). (**c**) The 3D z-stack of melanin masks and the 3D automatic segmentation of the epidermis and its sub-layers are jointly used to estimate a 3D epidermal, SC or LED melanin density. By defining 12-thickness normalized epidermal layers, the epidermal melanin density z-distribution (z-profile of melanin density in the 12 thickness-normalized epidermal layers from 1—DEJ level to 12—SC level) can be assessed.

The global 3D analysis of z-stacks of combined 2PEF-FLIM (4 time channels)/SHG images was performed with MPSTS software^50^ to identify skin layers, characterize DEJ 3D-shape and extract quantitative parameters on skin constituents and layers (Fig. 2; see Supplementary materials and methods).

### Statistical analysis

The results are mainly given in the text as estimated mean and standard deviation of the mean and the data distributions are described using histograms or box plots. Clinical trials data have been interpreted using both effect size (ES) and inferential analysis as currently recommended^56^. The data number per group is twice the number of volunteers as the two adjacent 3D z-stacks acquired per volunteer were considered separately.

## Results

### Comparison of FLIM and Pseudo-FLIM methods for melanin detection in different *in vitro* samples

**Figure 3:**
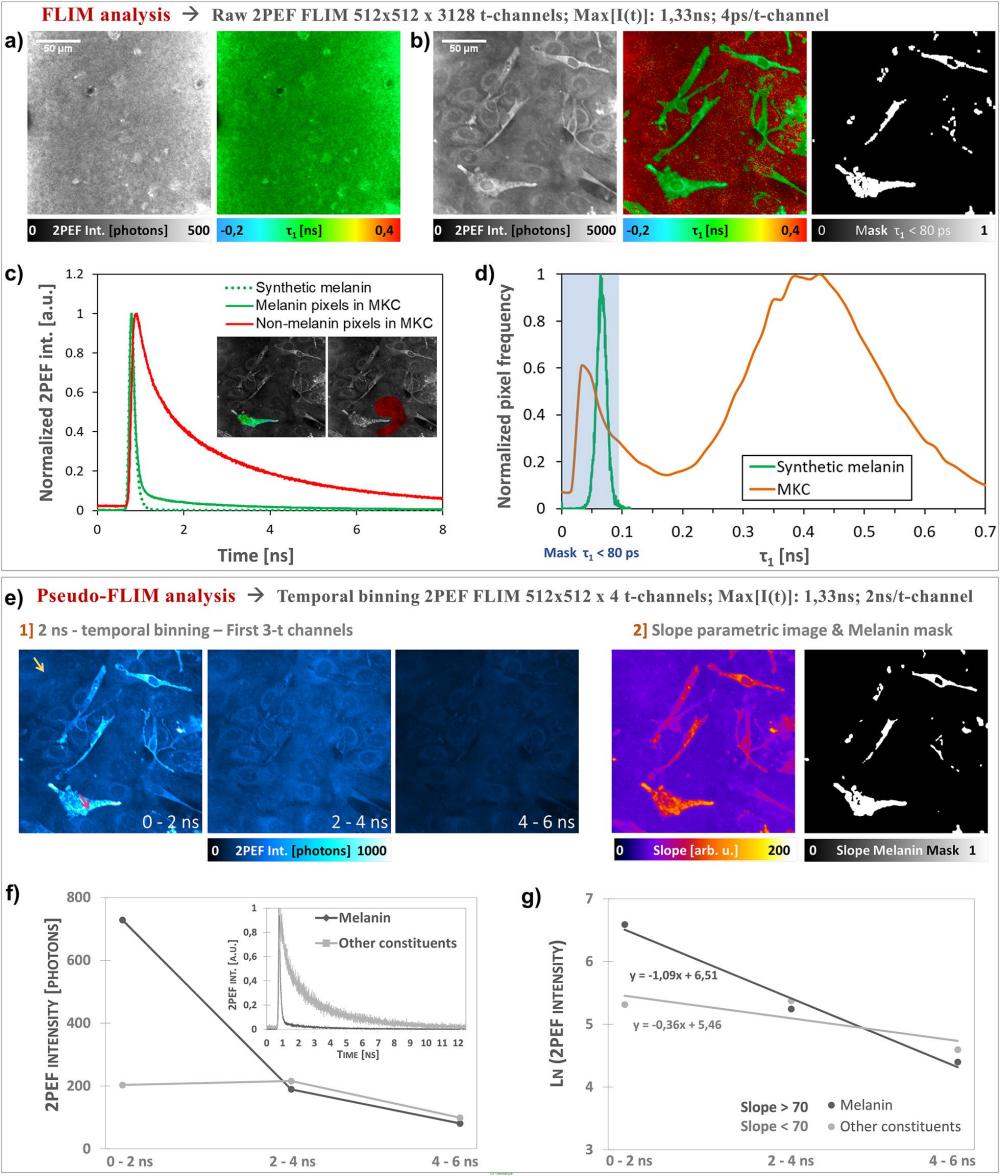
Comparison of FLIM bi-exponential fitting and Pseudo-FLIM methods for melanin detection. FLIM analysis – 2PEF intensity and τ_1_ short-fluorescence lifetime images of (**a**) synthetic melanin and (**b**) normal human skin melanocytes and keratinocytes NHMK coculture. The FLIM τ_1_ – based melanin mask is obtained by application of a threshold to keep the pixels with τ_1_ values below 80 ps. (**c**) 2PEF intensity decays of synthetic melanin, melanin and non-melanin pixels within the NHMK coculture (averaged 2PEF intensity within the green and respectively red regions of interest). (**d**) Normalized histograms of the pixel frequency for ${\tau }_{1}$ FLIM images of synthetic melanin (**a**) and NHMK coculture (**b**). The blue rectangle depicts the histogram regions selected by the application of a τ_1_ < 80 ps threshold. (**e**) Pseudo-FLIM analysis of NHMK coculture, involving (1) a temporal binning of the 2PEF decay into a reduced number of time channels with 2 ns integration time per channel, followed by (2) an estimation of the slope of the decay after linear regression on the first 3 t-channels. (**f**) Example of resulting binned 2PEF decay of a melanin pixel (red arrow) and a non-melanin pixel (yellow arrow) selected based on their τ_1_ value. (**g**) After a natural logarithm transformation, a linear regression of the first 3-time channels is performed to calculate the slope of the decay. The faster the decay, the higher the slope, as observed for these two melanin and non-melanin pixels. The image of the Pseudo-FLIM slope parameter in (**e**) is further processed for melanin detection to keep the high slopes values above 70 arb. u. and create a Pseudo-FLIM melanin mask.

Two-photon excited fluorescence intensity decays of synthetic melanin and normal human melanocytes/keratinocytes (NHMK) coculture were analyzed with FLIM and Pseudo-FLIM methods (Fig. 3). As expected, FLIM analysis allowed evidencing that synthetic melanin samples (Fig. 3a) have a short fluorescence lifetime ${\tau }_{1}$ of 66 ± 14 ps (see also its histogram in Fig. 3c) with a main relative contribution ${a}_{1}\left[\%\right]$ of 99 ± 2% and a long fluorescence lifetime ${\tau }_{2}$ of 1.59 ± 1.82 ns. These values are statistics over all the pixels within 5 images of 205 × 205µm^2^ (5 × 512 × 512 pixels) acquired at different locations within the synthetic melanin solution. This type of melanin is characterized by a fast decay, as shown in the histogram of photons arrival times in Fig. 3c and Pseudo-FLIM analysis yields indeed high slope parameter values of 178.75 ± 9.69 arb. u. We found similar fast decays and high slopes for natural melanins such as *Sepia* melanin and excreted melanin in 2D melanocytes cultures (data not shown). In NHMK coculture, we found two sets of pixels (e.g. for Fig. 3b: ${\tau }_{1}$=53 ± 14 ps; ${a}_{1}\left[\%\right]$=96 ± 3%; ${\tau }_{2}$=2.31 ± 0.64 ns and respectively ${\tau }_{1}$=419 ± 148 ps; ${a}_{1}\left[\%\right]$=60 ± 9%; ${\tau }_{2}$=2.73 ± 0.30 ns, statistics over the pixels in Fig. 3b) characterized by either (i) high slopes and FLIM lifetimes and relative contributions parameters similar to synthetic melanin or (ii) small slopes and FLIM parameters similar to free NAD(P)H and bound FAD and respectively bound NAD(P)H and free FAD (see Fig. 3b, d and e). The fluorescence lifetimes of NAD(P)H and FAD are exquisitely sensitive to enzyme binding during the cycling of the electron transport chain: protein-bound NAD(P)H lifetime is significantly longer than the free NAD(P)H lifetime, due to self-quenching in the free state while the FAD lifetime is short and long in the protein-bound and free states, respectively^57–59^. We recently demonstrated in unpigmented reconstructed human skin that single wavelength excitation at 760 nm enables both NAD(P)H and FAD imaging in keratinocytes, but the resulting 2PEF FAD intensity is low due to the disparities in the coenzymes’s concentration^60^, thus the 2PEF intensity signal at 760 nm in our cell samples mainly arises from NAD(P)H and melanin. Figure 3d shows the histogram of ${\tau }_{1}$ with values up to 200 ps mostly detected in melanocytes and in some pixels within keratinocytes, indicating the melanin transfer from melanocytes to keratinocytes. These values are in agreement with the reported ${\tau }_{1}$ values for melanin containing samples such as Dopa melanin and black hair^39^, melanocytes investigated in melanocytic nevi and melanomas^40^, melanocytes in a 3D skin model, human skin basal layer and hair bulb^41^ or synthetic melanin, melanocytes/keratinocytes coculture and human skin^38^. Pseudo-FLIM analysis of NHMK coculture is detailed in Fig. 3e, f and g. The fast decays, fast decrease in melanin 2PEF signal intensity with time is clearly visible on the first 3 t-channels images with 2 ns temporal binning (Fig. 3e). In Fig. 3f we have plotted both the raw decays (inserted graph) and the temporally binned decays for 2 pixels chosen within respectively melanocytes and keratinocytes cells. These pixels were attributed to melanin and respectively other endogenous constituents (NAD(P)H and FAD) based on their ${\tau }_{1}$ values and present different slope values (see Fig. 3g).

Based on these findings, we sought to define thresholds for FLIM short-fluorescence lifetime and Pseudo-FLIM slope parameter that will enable melanin detection in these samples. For synthetic melanin, based on the histogram in Fig. 3d, a ${\tau }_{1}$<80 ps threshold will allow selecting all melanin pixels. This type of synthetic melanin characterized by a predominant (~ 99%) and very short (< 80 ps) fluorescence lifetime and a long fluorescence lifetime (~ 1.59 ns) results in a very fast 2PEF intensity decay (Fig. 3c), corresponding in Pseudo-FLIM analysis to very high slopes values. Thus, for this type of “pure” melanin sample, a Pseudo-FLIM melanin mask could be obtained by applying a threshold on the slope parametric image to keep the slopes above 100 arb. u. In the melanocyte/keratinocyte coculture, the FLIM parameters are slightly different compared to the ones of synthetic model melanin and this is expected as natural melanin is a mixture of eu-/pheo-melanin^6^. Modulations in these ${\tau }_{1}$, ${\tau }_{2}$, ${a}_{1}\left[\%\right]$ or ${a}_{2}\left[\%\right]$ parameters will be reflected on the shape of the 2PEF intensity decay and thus on the steepness of the slope of the decay. This is clearly seen in Fig. 3c for the melanin containing pixels in the melanocyte ($\tau_{1}$~ 53 ps, $a_{1} \left[ \% \right]$~96% and $\tau_{2}$~2.3 ns). The changes in these parameters, and mainly the increase in $\tau_{2}$, results in slightly slower decays, corresponding to smaller Pseudo-FLIM slope values as compared to synthetic melanin. Accordingly, the threshold value for Pseudo-FLIM slope parameter must be decreased. We found that by applying a threshold to keep the high slope values above 70 arb.u., the Pseudo-FLIM melanin masks highlights almost the same pixels as the ones in the FLIM ${\tau }_{1}$<80 ps melanin mask (Fig. 3a and e). These data allow demonstrating that Pseudo-FLIM analysis can identify melanin pixels based on the slope of their 2PEF intensity decay in synthetic melanin and melanocytes keratinocytes coculture.

### Comparison of FLIM, phasor and Pseudo-FLIM methods for melanin detection in human skin *in vivo*

**Figure 4:**
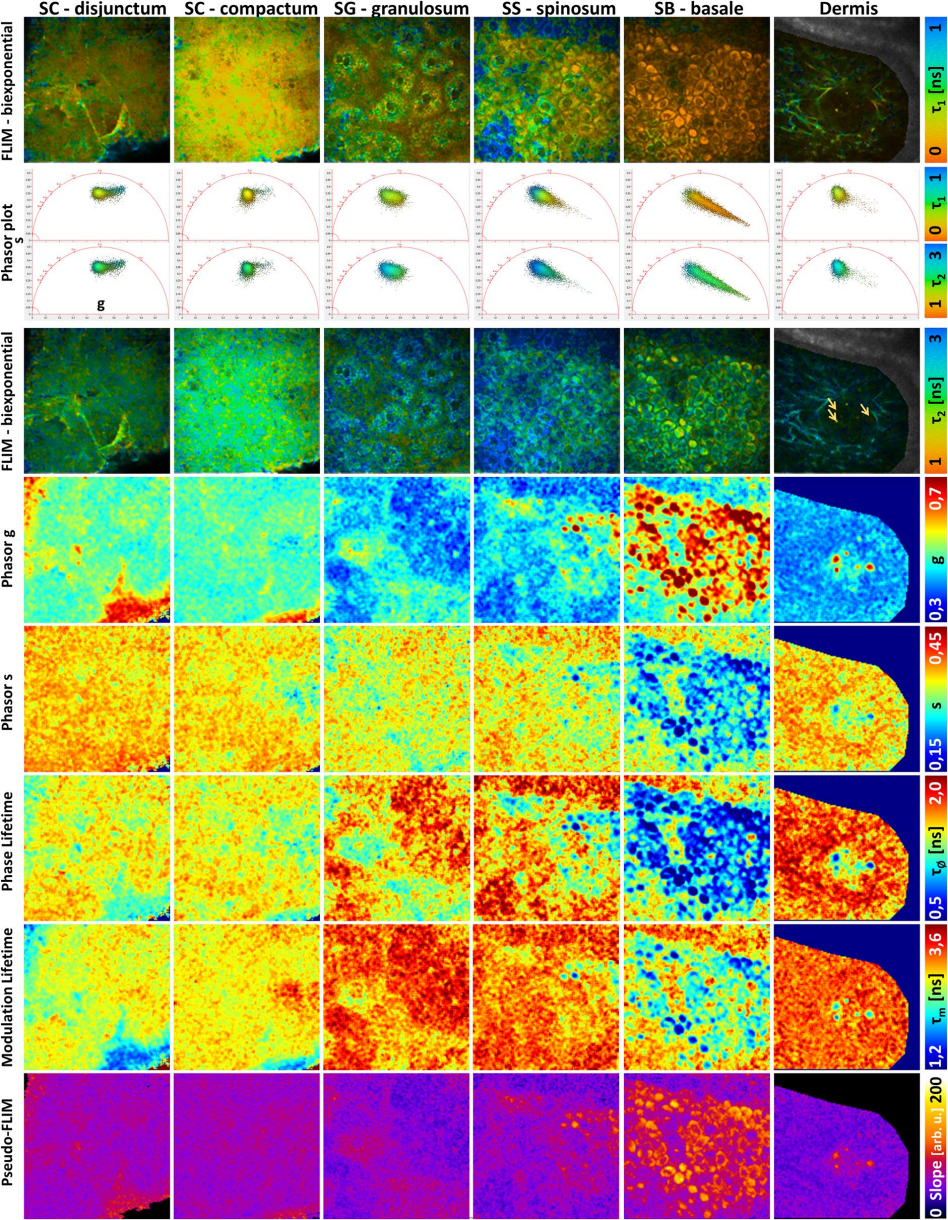
Comparison of FLIM bi-exponential fitting, Phasor and Pseudo-FLIM methods for melanin detection *in vivo* on human skin. Multiphoton 2D 2PEF FLIM images acquired at different depths from the skin surface to the dermis, within *stratum cornuem* (SC) *disjunctum*, *corneum compactum*, *granulosum* (SG), *spinosum* (SS), *basale* (SB) and superficial dermis were analyzed with the three methods. (top) FLIM bi-exponential fitting analysis images of the short τ_1_ and long τ_2_ fluorescence lifetime parameters. (middle) Phasor analysis images of g, s, phase lifetime and modulation lifetime parameters. The corresponding phasor plots (s versus g scatters) of the different skin layers are inserted in between the τ_1_ and τ_2_ images and color coded using the same color scale as for τ_1_ and τ_2_ parameters. An enlarge view is shown in Fig. S2. (bottom) Pseudo-FLIM analysis images of the slope parameter highlighting pixels with a fast decay. The arrows indicate the fast decay pixels within the blood capillary.

Next, we sought to determine if the same threshold for Pseudo-FLIM melanin mask defined in the NHMK coculture also works for *in vivo* human skin. We have acquired 2D 2PEF FLIM images at different depths from the skin surface to the dermis, within *SC disjunctum*, *SC compactum*, *granulosum*, *spinosum*, *basale* and superficial dermis and analyzed the data with FLIM bi-exponential fitting, phasor and Pseudo-FLIM methods (Fig. 4, Fig. S2). Our imaging conditions (760 nm excitation, broad spectral detection 390–650 nm) were chosen to detect the endogenous autofluorescence of skin constituents such as NAD(P)H, FAD, melanin, keratin and elastin^35,36,42^. The diversity in skin constituents and mixture of constituents at the pixel level is clearly seen in the phasor plots patterns of the different layers and bi-exponential fitting images. The histograms of parameters shown in Fig. 4 and their average values within different skin layers is given in Fig. S3.

Living cells such as non-melanized keratinocytes of the basal, spinosum and granulosum layers have fluorescence signals dominated by NAD(P)H’s emission in our imaging conditions^61,62^. Although FAD has a larger two-photon absorption cross section than NAD(P)H at 760 nm^61^, its low fluorescence quantum yield and the disparities in NAD(P)H and FAD coenzymes’s concentration lead to a low 2PEF FAD intensity, as measured in living keratinocytes within unpigmented reconstructed human skin^60^.The cells of *stratum granulosum* in Fig. 4 show a phasors’s pattern and bi-exponential analysis parameters equivalent to the ones of living keratinocytes within unpigmented reconstructed human skin^60,63^.

Depending on the constitutive skin pigmentation, keratinocytes of the basal and supra-basal layers also contain different amounts of melanin within the melanosomes transferred from the melanocytes. Some melanin, may also be found in corneocytes, within non-degraded melanosomes. In normal human skin, melanocytes are found within *stratum basale*, but they cannot be identified in multiphoton FLIM based on their melanin content like in melanoma^40,64^ or in a 2D cellular culture (e.g. Fig. 3). Indeed, in healthy skin, fully melanized type IV melanosomes are found at the tips of their dendrites and are directly transferred to keratinocytes. Melanized keratinocytes have a shorter ${\tau }_{1}$ fluorescence lifetime (Fig. 4) and a higher relative amplitude ${a}_{1}\left[\%\right]$ (Fig. S2) compared to non-melanized keratinocytes with mainly NAD(P)H as a fluorescent constituent (e.g. ${\tau }_{1}$ and ${a}_{1}\left[\%\right]$ histograms in Fig. S3). These results are in agreement with human skin basal layer data published by Dancik et al*.*^38^. The shortest ${\tau }_{1}$ and highest ${a}_{1}\left[\%\right]$ values were mainly found within melanized keratinocytes in *stratum basale* and also within some keratinocytes and corneocytes within other skin layers. The phasor plots of *stratum spinosum* and *basale*, with a comet like pattern pointing towards a very short lifetime component around 0.1 ns, are a mixture of different lifetime components (mainly free/bound NAD(P)H, to a less extent free/bound FAD and melanin (short and long lifetimes)). Similar phasor pattern was also evidenced in human choroidal melanocytes (NADH/melanin constituents)^43^. The signal intensity was not high enough to allow for multi-exponential analysis, so the data were only approximated using a bi-exponential decay function. Pixels with high g and small s values, corresponding to very small phase lifetimes, mainly contain melanin.

Pixels with high g and small s values, short phase lifetime, were also found within the capillary vessels of the dermis. In the example of Fig. 4, we detected a very short and predominant fluorescence lifetime component similar to melanin, probably arising from hemoglobin within the red blood cells^65–67^. Elastin within elastic fibers has longer phase lifetimes compared to melanin and a phasor plot overlapping with the one of non-melanized keratinocytes.

In *SC*, pixels with ${\tau }_{1}$ values around 250 ps and respectively 800 ps were found (Fig. S3). The long lifetime values could translate either the keratinization process or melanin degradation. We don’t know how the natural process of melanin degradation in the upper skin layers impacts its molecular structure and organization and thus its fluorescence lifetime. The phasor plot pattern is also different compared to other layers: a round shape pattern shifted towards higher g values, although partly overlapping the phasors of non-melanized keratinocytes and a comet like pattern probably corresponding to more keratinized cells. The pixels at the tip of the comet are found within the uppermost corneocytes at the air/skin interface and are characterized by a major contribution of ~ 800 ps lifetime component. This phasor pattern is however different from the reported hair keratin phasor plot^42,43^ suggesting different fluorescence properties of keratinized hair and skin tissues. Whether these pixels correspond to “more keratinized” structures or to degraded melanin it is unknown, and this will require further analyses, beyond the scope of this paper.

Pseudo-FLIM analysis highlights pixels with high slope values corresponding to fast 2PEF intensity decays. The images in Fig. 4 show that these high slope values pixels are also characterized by small ${\tau }_{1}$, high ${a}_{1}\left[\%\right]$, high g, small phase and modulation lifetimes values. Given all these skin characteristics, the question is how to quantify melanin from multiphoton FLIM data? We applied different thresholds to create melanin masks (Fig. S4): ${\tau }_{1}$<100 ps, ${\tau }_{1}$<150 ps, ${a}_{1}\left[\mathrm{\%}\right]$>90%, ${\tau }_{AvAmp}$<400 ps, g > 0,5 & s < 0.3 (to keep the melanin containing pixels and remove the non-melanized pixels and the pixels within the comet pattern of the *stratum corneum*) and ${\tau }_{\varphi }$<1,1 ns. By applying a threshold to keep the high slope values above 70, we created a Pseudo-FLIM melanin masks similar to the melanin masks of amplitude weighted average lifetime (bi-exponential analysis) and phase lifetime (phasor analysis). Pseudo-FLIM mask includes the pixels within the ${\tau }_{1}$<100 ps and ${a}_{1}\left[\%\right]$>90% masks but also includes other pixels. By increasing the threshold value of the short-fluorescence lifetime to 150 ps, more pixels with smaller relative contributions are identified, but they are not selected by the other parameters.

Within SC, it seems that the slope > 70 arb. u. threshold also allows identifying pixels with high g values, within the comet pattern. We also compared Pseudo-FLIM analysis (slope estimation upon linear regression on the first 3 temporally binned 2 ns time-channels, 1,33 ns peak position)^48,49^ with an analysis based on the subtraction of two overlapping temporally binned time-channels^68^ that is a linear regression on two channels, corresponding to a Pseudo-FLIM analysis on two time-channels. The images (data not shown) were quite similar, and we observed that this 2-channel method also detects the same pixels with high g values, ${\tau }_{1}$~800 ps, ${a}_{1}\left[\%\right]$~70%, within the comet pattern of SC.

The comparison of FLIM bi-exponential, phasor and Pseudo-FLIM analyses allows confirming that *in vivo* melanin pixels characterized by ${\tau }_{1}$<100 ps, ${a}_{1}\left[\%\right]$>90%, g > 0.5 & s < 0.3 are detected by Pseudo-FLIM analysis, which also includes other pixels with ${\tau }_{\varphi }$< ~ 1.1 ns. Based on these considerations, melanin detection by Pseudo-FLIM seems to be specific within the living epidermis, but some fast decay pixels within some regions of the uppermost cells of SC are also detected.

### Pseudo-FLIM melanin analysis is compatible with 3D imaging in clinical trials

**Figure 5:**
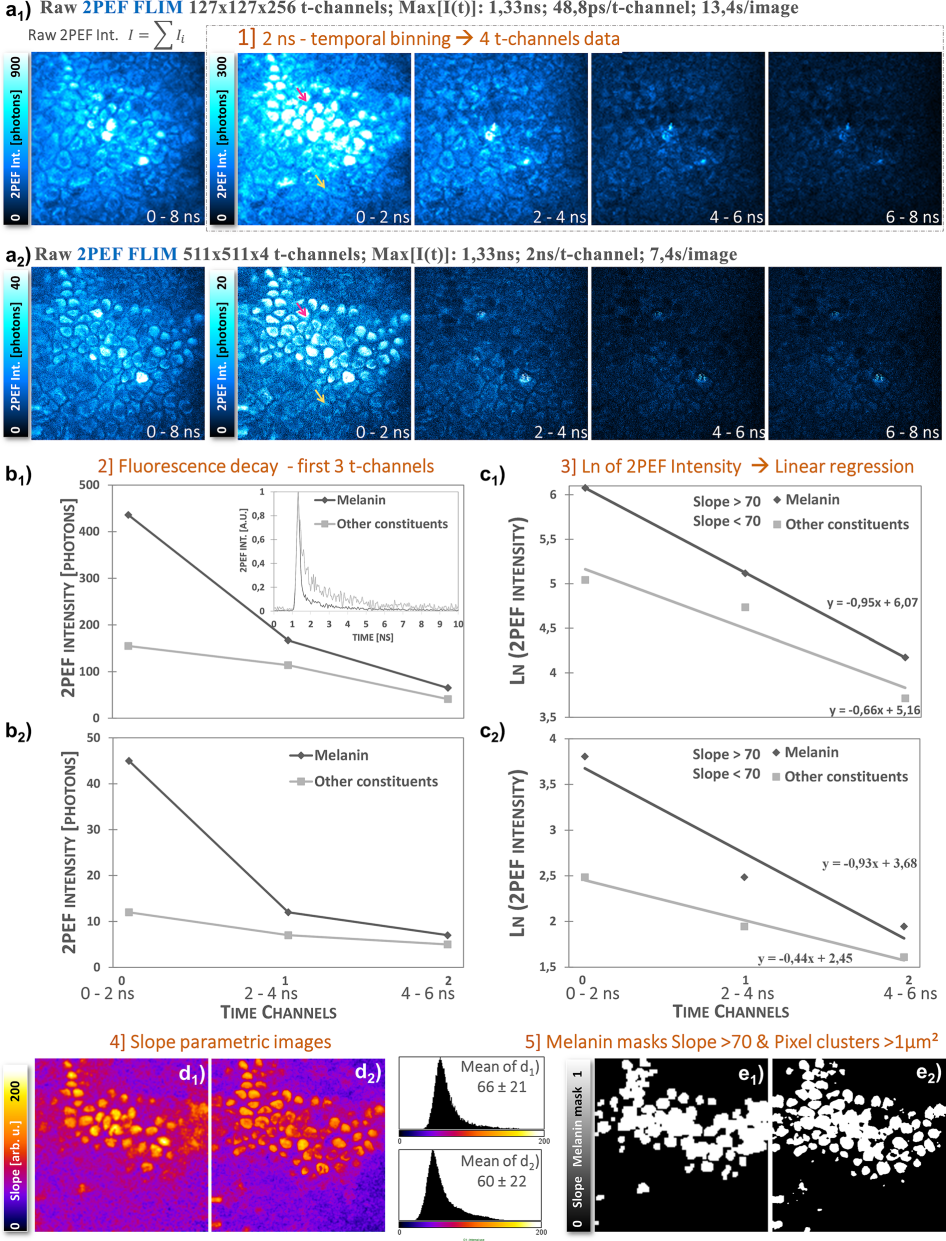
Effect of image acquisition time on *in vivo* human skin Pseudo-FLIM melanin detection. (top) 2PEF intensity images within 0–8 ns time range and within the first 4 temporally binned time channels with 2 ns integration time. (**a1**) 2D 2PEF-FLIM image of 127 × 127 pixels × 256 time channels acquired within the basal layer of the epidermis (104 µs pixel dwell time); (**a2**) 2D 2PEF-FLIM image of 511 × 511 pixels × 4 time channels extracted from a z-stack and acquired almost within the same 2D plane at 28 µs pixel dwell time. (**b1**, **b2**) shows the corresponding 2PEF intensity decays of the first 3 temporally binned channels for a high slope (red arrow, melanin) and small slope (yellow arrow, other constituents) pixels. The full decay of these two pixels extracted from the 256 t-channels images is given in the insert in graph (**b1**). Graphs (**c1**, **c2**) show the natural logarithm transformation and linear regression fitting to extract the slopes of the decays. Images (**d1**, **d2**) show the corresponding slope parametric images and their Pseudo-FLIM melanin mask is given in images (**e1**, **e2**).

We first acquired an *in vivo* multiphoton z-stack of combined 2PEF-FLIM (4 time channels)/SHG images with 28 µs pixel dwell time (Fig. 5b). We chose afterwards a 2D plane within the basal layer and acquired a 2PEF-FLIM image (256 time channels) with decreased spatial resolution (4× spatial binning of 511 × 511 → 127 × 127 pixels) and increased image acquisition time (104 µs pixel dwell time) (Fig. 5a). The two images were acquired almost within the same z 2D plane of the basal layer (a little shift in z can be perceived between images). The comparison of 2 ns temporally binned images clearly shows that the high intensity melanin signals are mostly decaying within the first 2 ns time-window. Figure 5b1 and b2 show 2PEF intensity decays for a melanin pixel (fast decay, high slope value) and a “other constituents” pixel (slower decay, small slope value). After natural logarithm transformation and calculation of the linear regression (Fig. 5c1 and c2), we obtained almost the same slopes for melanin pixels. The slope parametric images, their histograms (Fig. 5d1 and d2) and corresponding melanin masks (Fig. 5e1 and e2) are quite similar given the slight z-difference between the two images and the spatial resolution differences.

**Figure 6:**
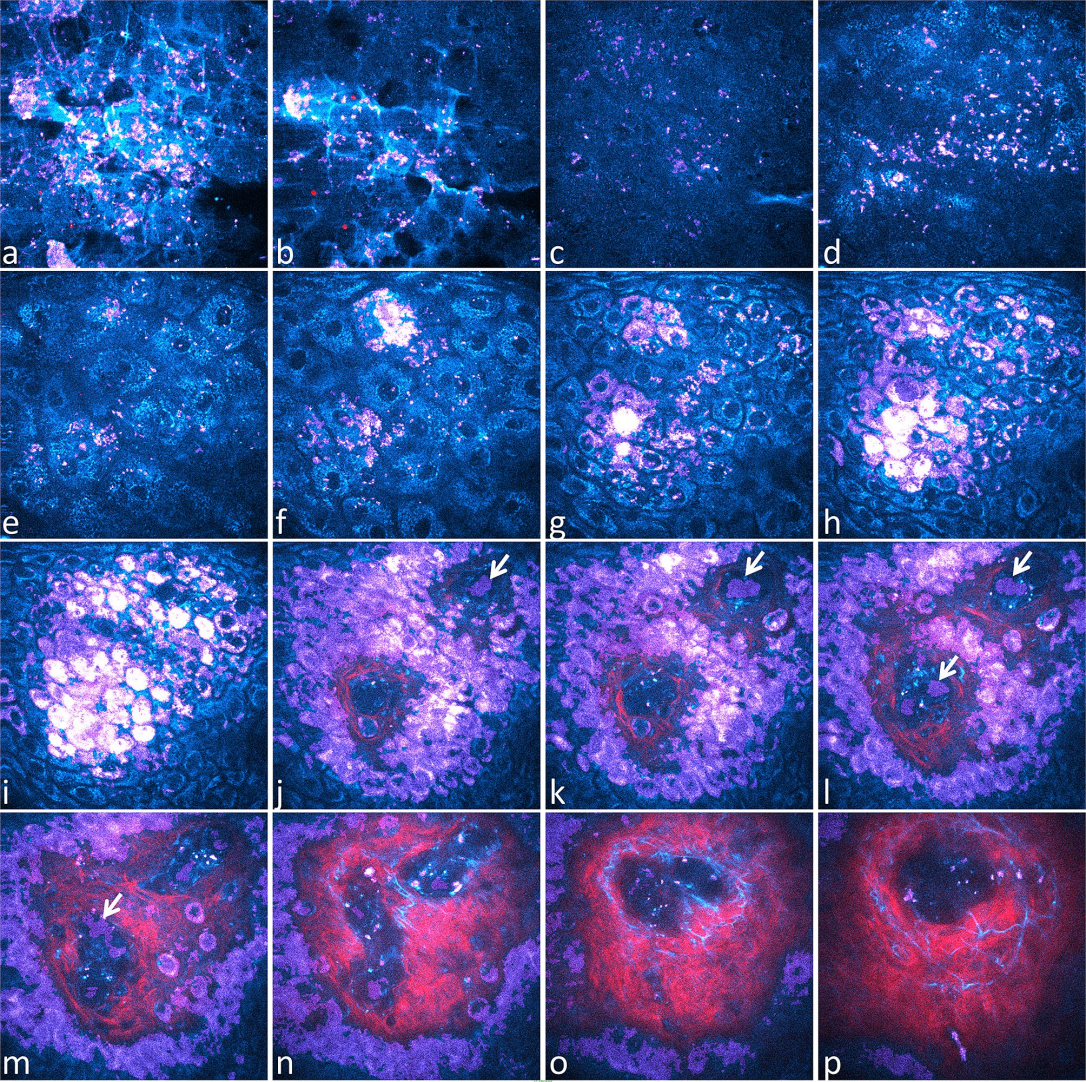
*In vivo* 3D multiphoton images of human skin – acquisition of combined 2PEF-FLIM (4 time channels)/SHG z-stacks compatible with Pseudo-FLIM melanin detection. 2PEF intensity is shown in cyan hot color, SHG in red and Pseudo-FLIM melanin mask pixels in purple. High 2PEF signal intensities appear in white color. Images are extracted from a z-stack of 70 *en face* images acquired  with 2.346 µm z-step. (**a**) *stratum corneum disjunctum*; (**b**) *stratum corneum disjunctum/compactum* interface; (**c**, **d**) *compactum* / *granulosum* interface; (**e**, **f**) *stratum granulosum*; (**g**, **h**) *stratum spinosum*; (**i**) *stratum basale*; (**j**–**o**) *stratum basale*/dermis interface; (**p**) superficial dermis. Within the blood capillaries, Pseudo-FLIM detects cells with high slope values, fast decays, that most probably emit 2PEF signals from hemoglobin (see arrows). As the image acquisition time is slower than the blood flow, some blood cells appear with a deformed shape as indicated by the arrow in image m.

We can conclude that Pseudo-FLIM melanin detection is still feasible upon decreasing the image acquisition time and opens the possibility to quantify melanin from 3D z-stacks. An example of Pseudo-FLIM melanin detection from 3D multiphoton clinical data is given in Fig. 6. In this z-stack, one clearly can see that Pseudo-FLIM not only detected high 2PEF intensity pixels of basal keratinocytes but also pixels with lower 2PEF intensity but fast decays.

### Applications of Pseudo-FLIM *in vivo*: modulation of melanin global 3D density and z-epidermal distribution upon different conditions

Measurement of melanin 3D density was performed in the entire epidermis (global density) and into several thickness-normalized epidermal sub-layers (z-distribution), allowing distinguishing its neosynthesis or inhibition in the basal layer versus modulations of its distribution in the upper layers. Changes in melanin density in epidermal sub-layers can impact or not the global density. For example, a decrease in one layer could be counteracted by an increase in another layer, and hence, no change will be evidenced in the global density. It also allows evidencing slight modulations that are not detectable at the global level^52^.

### Constitutive pigmentation (original data)

**Figure 7:**
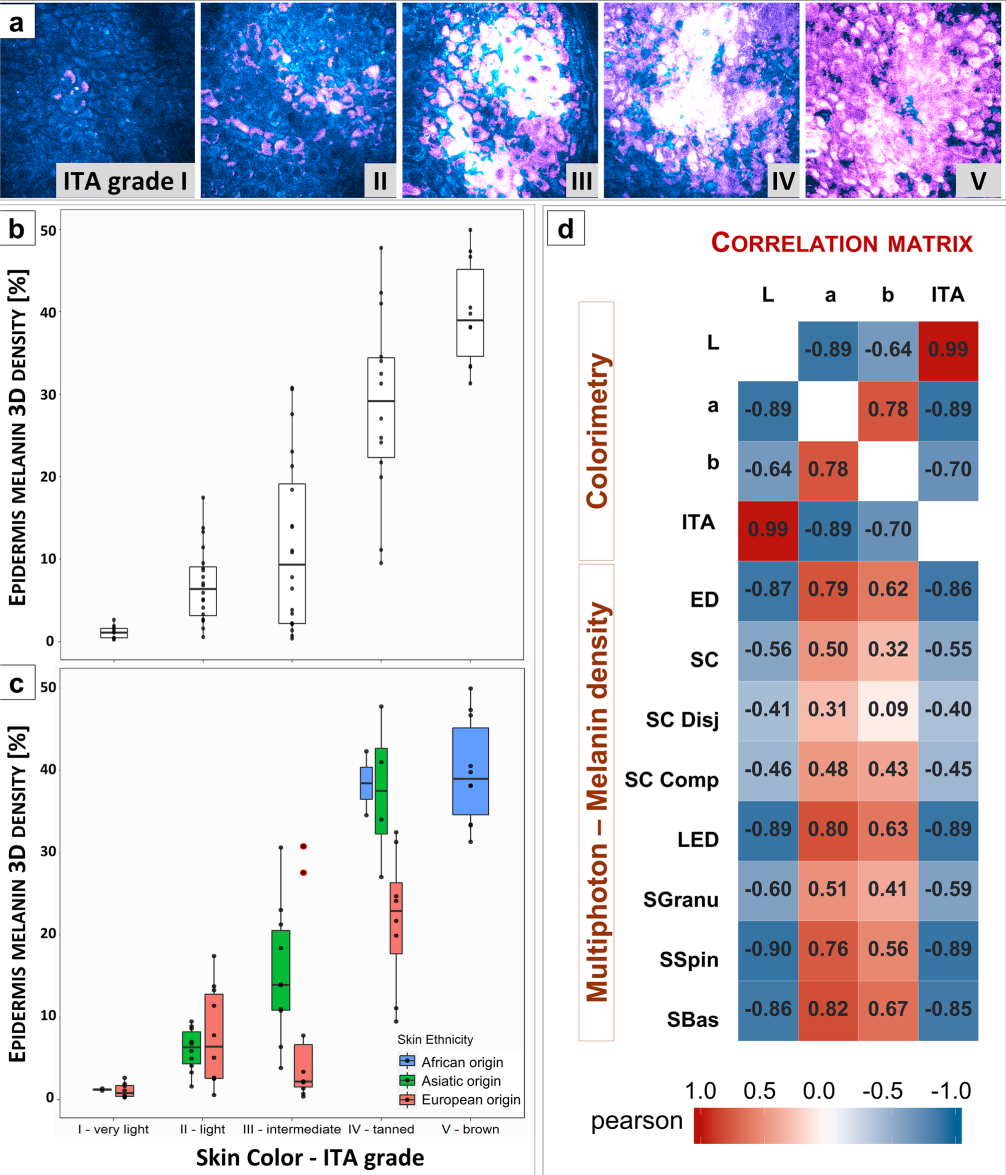
Modulation of global epidermal 3D melanin density with skin color ITA grade in constitutive pigmentation study. (**a**) Raw 2PEF intensity images (cyan hot) and Pseudo-FLIM melanin masks (purple) acquired within the basal layer of human forearm skin. High signal intensities appear in white color. (**b**) Modulation of 3D epidermal melanin density with ITA group and (**c**) with both ITA group and skin ethnicity. The data are expressed as boxplots with fences. (**d**) Correlation matrix of the skin colorimetric parameters and multiphoton melanin density estimated in the epidermis and its sub-layers. The data correspond to the Pearson correlation coefficients. An absolute value of the Pearson correlation coefficients between [0.6, 0.8] and [0.8, 1.0] indicate a strong and respectively a very strong correlation. ED (global epidermis), SC - *stratum corneum*, SC Disj - *stratum corneum disjunctum*, SC Comp - *stratum corneum compactum*, LED – living epidermis, SGranu - *stratum granulosum*, SSpin – *stratum spinosum* and SBas - *stratum basale*.

Combined 2PEF intensity and Pseudo-FLIM melanin masks images allow observing the changes in melanin density with skin color ITA groups (Fig. 7a). As expected, global 3D epidermal melanin density progressively increases in the lightest to darkest skin color groups (Fig. 7b, c, Table S1) and is highly correlated with skin ITA value (negatively, Fig. 7d) in agreement with ex vivo results on skin samples with variable constitutive pigmentation^9^. Although the correlations are strong, the link between skin color and melanin content is not necessarily linear: indeed, in group III, for the same measured color, the global melanin density is clearly higher in the subgroup of Asian origin (Fig. 7c). Such difference is not observed between Eu.O and As.O subjects in group II, possibly due to the very low density of melanin in light skin-type groups. Clear differences in global melanin density between Asian and European volunteers in groups III and IV (Fig. 7c) are associated with small ones for the L*, b* and ITA parameters (Fig. S5). The Asian volunteers have a slightly darker skin color and an increased b* value representing the blue–yellow component.

Although partly explainable by these different contributions of colorimetric parameters probably due to a different melanin composition, the significant differences in global melanin density between Asians and Europeans for a same ITA group suggest that skin color also depends on other factors as the distribution of the pigment in the thickness of the layer (see below) or on the morphology and organization of melanosomes.

**Figure 8:**
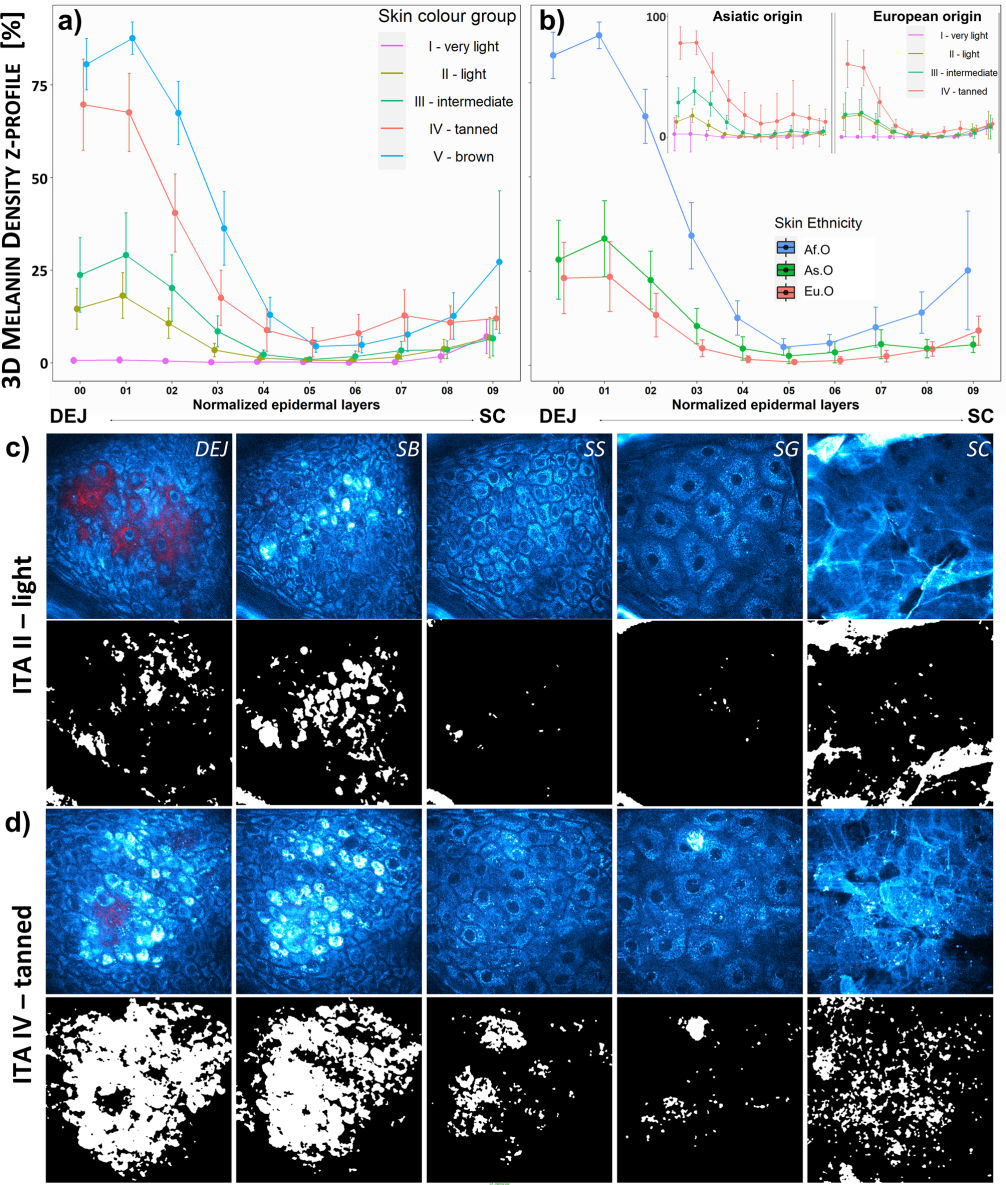
Modulation of z-epidermal distribution with skin color ITA grade and skin ethnicity in constitutive pigmentation study. Melanin density z-epidermal distribution profiles (mean 3D melanin density estimated in 12 thickness-normalized epidermal layers from 1—DEJ level to 12—SC level) of (**a**) different skin color ITA groups and b) different skin ethnicity. The insert in (**b**) is showing the z-profiles of different skin color ITA groups for Asiatic and European origins. The z-profiles data are expressed as mean ± confidence intervals of the mean. Raw 2PEF intensity images (cyan hot) and Pseudo-FLIM melanin masks (white) extracted from multiphoton z-stacks acquired on ventral forearms of volunteers with (**c**) light and (**d**) tanned skin color ITA groups. DEJ – dermal–epidermal junction level, SB - *stratum basale*, SS – *stratum spinosum*, SG - *stratum granulosum* and SC - *stratum corneum*.

Melanin density z-distribution profiles with skin color ITA group (Fig. 8a) and skin ethnicity (Fig. 8b) allow evidencing a progressive increase in melanin density at the basal and supra-basal layers for ITA grades II to V and a slight increase at the level of *stratum granulosum* and *stratum corneum* in tanned and brown skin. An example of images showing the melanin density z-differences between light and tanned skin is given in Fig. 8c, d. In group III, the epidermal melanin distribution is clearly different between Eu.O and As.O subgroups (see insert in Fig. 8b), with a significant melanin amount above the basal layer in the latter, lower however than observed in groups IV and V in which significant amounts of melanin are observed up to half of the epidermis, as well as an increase within SC (Fig. 8a). Mean epidermal thickness being comparable among ethnicities (Eu.O 60.5 ± 9.8 µm, As.O 65.1 ± 10.9 µm and Af.O 66.6 ± 5.4 µm), this confirms *in vivo* that constitutive pigmentation is not only given by melanin amount, but also by its epidermal distribution.

Higher melanin density but more distributed in Asians may also explain the better UV protection of Asian skins, even light ones, compared to European skins.

Overall, the small amount of melanin detected within SC, including in the darkest skins, shows that it has little contribution to the global density, confirming that the fast decay pixels detected in some regions within the uppermost cells of SC*,* whether they correspond to “more keratinized” structures or to degraded melanin, have little impact on the global epidermal melanin density parameter (Fig. S6).

### Chronic and seasonal sun exposure (original and published^52^ data)

The data for chronic exposure (Fig. S7) are original data from photo-aging study comparing ventral and dorsal (mostly unexposed vs. exposed) forearms. The data for seasonal sun exposure (Fig. S9) are from retinoids 1 year-study and correspond to acquisitions performed at months M00 (March), M03 (June), M06 (September), M12 (March + 1 yr) on the control untreated forearm of volunteers; these have already been published^52^).

Melanin density is obviously higher on chronically exposed zones (dorsal versus ventral forearm both in young and aged subjects, without differences between groups for a same site) (Fig. S7) and it increases progressively with summer in the 1 year-study (Fig. S9a).

Analysis of melanin z-distribution in photo-aging study shows an increased melanin density in basal and supra-basal layers in aged versus young subjects (Fig. S7f).

Among seasonality (1 year-study, control condition), the gradual increase of global density is not only due to an increase in the basal layers, but also up to the middle epidermis at month M03, and throughout the epidermis at month M06 after summer (Fig. S9c). After 1 year, a persistent accumulation of melanin in supra-basal layers, as well as at the SC level is observed when compared to M00. Thus, the progressive accumulation of melanin in the supra-basal layers of the epidermis could be part of the pigmentary changes that occur during aging and the melanin distribution profile could be a new pertinent descriptor of photoaging at least in Europeans.

### Treatment (published in^51,52^)

In the study of retinoids effects under occlusion^51^, a clear decrease of melanin content in the living epidermis is evidenced on retinol-treated area (Fig. S8), whereas no significant modification is observed at the global level after one year of usual application conditions^52^ (Fig. S9a). In this latter case, in retinol-treated group (Fig. S9c), although the strongest modulation of melanin distribution induced by summer (M06) isn’t different compared to control group, the ascent of melanin in supra-basal layers, appearing in the control group, isn’t observed at months M03 nor at M12. The melanin z-distribution at these times overlaps M00 curve for retinol-condition, strongly suggesting that in vivo, the predominant effect of retinoids on pigmentation is more likely due to skin renewal than to a direct effect on melanogenesis.

## Discussion and conclusions

In this work, we implemented a 3D melanin detection approach called Pseudo-FLIM, based on slope analysis upon linear regression of temporally binned two-photon excited autofluorescence lifetime data^48,49^. We demonstrated by comparison with FLIM bi-exponential and phasor analyses that the fast decay shapes of melanin containing samples correspond to high slope values, a characteristic one can use to identify melanin containing pixels *in vitro* or *in vivo*. To our knowledge, this is the first time that phasor approach was applied to melanin analysis *in vivo* on human skin and that FLIM bi-exponential and phasor methods were compared for melanin detection. The long image acquisition time needed for acquiring enough photons per pixel for bi-exponential or phasor analysis limits the application of these methods to selected 2D epidermal or dermal depths and, in practice, is not compatible with 3D skin imaging in a clinical trial. Using Pseudo-FLIM approach, one can perform a 3D melanin detection from an entire z-stack of *in vivo* multiphoton images of human skin as well as other types of melanized samples. In association with an automatic 3D epidermis segmentation^50^, a global 3D epidermal melanin density can be estimated. Additionally, as the epidermal thickness may vary according to the anatomical sites, skin ethnicity, aging or products application, we introduced a more refined segmentation of the epidermis that allows extracting a melanin z-epidermal distribution profile and comparing skin regions with varying epidermal thickness.

*In vivo* multiphoton 3D imaging in human volunteers has limitations in terms of image field of view (~ 130 × 130 µm^2^ for DermaInspect), but the investigated volume is higher compared to histology 2D FM analysis^9,10,11^ (usually a few images of ~ 200–250 µm epidermal length are analyzed within a skin biopsy) and 2 times higher field of views can be achieved with systems such as MPT*flex*. Despite the small investigated epidermal volume, we have demonstrated that multiphoton global 3D epidermal melanin density is highly correlated with skin ITA value in constitutive pigmentation study, in agreement with *ex vivo* histology and HPLC chemical analysis results on skin samples with variable constitutive pigmentation^9^. Moreover, our results indicate that multiphoton imaging can provide quantitative data of interest in studying pigmentation modulations under different conditions (constitutive and acquired pigmentation, aging, natural UV exposure^52^ or application of topical retinoids^51,52^ or corticosteroids^45^), but its ability to accurately assess melanin heterogeneity in skin pigmentary disorders remains to be studied.

Multiphoton Pseudo-FLIM can address a wide range of *in vivo* situations and melanin’s epidermal distribution provides new and valuable information that complements melanin content; for example, it can probably explain the difference in perceived color and photosensitivity between Asian and European skins though very similar colorimetric values. It also makes it possible to study treatments’ mechanism of action, as illustrated with retinoids, whose action on melanin is due to epidermal renewal and not to a direct effect on melanin as discussed in the literature^52^. In another case, the progressive accumulation of melanin in the supra-basal layers of the epidermis observed during aging probably explains in part the pigmentary changes that occur over time; the demonstration of its reversion under retinoids, the gold standard of anti-aging, validates this parameter as a relevant descriptor of photoaging in Europeans.

This method could also bring new insights into the knowledge of some underlying biological mechanisms of pigmentation modulations appearing through either redistribution of existing melanin and/or *de novo* melanin synthesis. A still challenging topic, especially in photoprotection, is the *in vivo* characterization of melanin modulations upon UV exposure and their link to the clinical manifestations of hyperpigmentations, i. e. Immediate Pigment Darkening (IPD) and Persistent Pigment Darkening (PPD). Assessing melanin’s global density and 3D distribution *in vivo* with multiphoton Pseudo-FLIM will avoid performing invasive biopsies and will certainly help understating the mechanisms of skin photobiology and how melanin is modulated with different UV wavelengths and doses. Knowing the importance of epidermal melanin distribution for its DNA protection factor^69–71^, we are confident that multiphoton Pseudo-FLIM approach will contribute to developing tomorrow’s photoprotection products. More generally, the *in vitro* and *in vivo* applications of multiphoton Pseudo-FLIM based melanin detection and quantification of its global 3D density and z-epidermal distribution encompass physiological, pathological, or environmental factors-induced pigmentation modulations up to whitening, anti-photoaging, or photoprotection products evaluation.

## Supplementary Information

Supplementary Information.
