# Supplementary material for: In vivo melanin 3D quantification and z-epidermal distribution by multiphoton FLIM, phasor and Pseudo-FLIM analyses
Source: Sci Rep. 2022 Jan 31;12:1642. doi: 10.1038/s41598-021-03114-0 (PMC8803839; doi:10.1038/s41598-021-03114-0)
Supplement: Supplementary file 1 — Supplementary Information. [file 41598_2021_3114_MOESM1_ESM.docx]

***In vivo* melanin 3D quantification and z-epidermal distribution by multiphoton FLIM, phasor and Pseudo-FLIM analyses**

Ana-Maria Pena^1*^, Etienne Decencière^2^, Sébastien Brizion^1^, Peggy Sextius^1^, Serge Koudoro^2^, Thérèse Baldeweck^1^, Emmanuelle Tancrède-Bohin^3,4^

^1^L’Oréal Research and Innovation, 1 avenue Eugène Schueller, BP22, 93601, Aulnay-sous-Bois, France.

^2^MINES ParisTech – PSL Research University, Fontainebleau, France

^3^L’Oréal Research and Innovation, Campus Charles Zviak RIO, 9 rue Pierre Dreyfus, Clichy, France.

^4^Service de Dermatologie, Hôpital Saint-Louis, Paris, France

*Correspondence to ana-maria.pena@rd.loreal.com

# Supplementary materials and methods

## *In vitro normal human melanocytes / keratinocytes (NHMK) coculture*

Normal human melanocytes (NHM) and keratinocytes (NHK) were grown in respectively K - LL-0007 and M -LL-0027 Cellsystems® Medium Complete Kit culture mediums and, at confluence, trypsinated and co-seeded (17 000 NHM, 50 000 NHK) in 96 wells plates in NHK growth medium. After 3 days, the cocultures were grown for 4 days in a depleted culture medium (3:1 volume ratio of Gibco DMEM 31053-028 and F12 31765-027). Before imaging, they were fixed with 200 µL of 4% formaldehyde solution and rinsed with 200 µl of PBS.

## FLIM bi-exponential analysis

FLIM bi-exponential analysis of *in vitro* (512 x 512 pixels x 3128 time channels) data was performed using SymPhoTime 64 (v2.1.3813, Picoquant, Berlin, Germany). Images were spatially binned to 256 x 256 pixels to increase the signal intensity per time channel and FLIM parameters were calculated using a 2-exponential reconvolution model and calculated instrumental response function (IRF).

FLIM bi-exponential analysis of *in vivo* (128 x 128 pixels x 256 time channels) data was performed using SPCImage (v8.1, Becker & Hickl, Berlin, Germany). The decay data within an area of 3x3 pixels (binning of 1), were binned together to increase the signal intensity per time channel and the accuracy of lifetime analysis. We used a two components weighted least squares model function with automatic IRF calculated from the rising edge of the decay data and a fixed shift parameter (initialized by the highest pixel intensity within an image).

For both *in vitro* and *in vivo* analyses, the 2PEF intensity and calculated images were exported in tif or asc format and further processed for melanin quantification using home-written macros with Fiji/ImageJ (W. Rasband, NIH, USA).

## FLIM phasor analysis

By applying a Fourier transform to every pixel, the 2PEF intensity decay is transformed into a phasor with coordinates *g* and *s* within the phasor plot (Figure 1b). The real *g* and complex *s* components of respectively cosine and sine transforms are defined by $g_{i,j}\left( \omega\right)=\frac{\int_{0}^{\infty} I_{i,j}\left( t \right)\cos\left( \omega t \right)dt}{\int_{0}^{\infty} I_{i,j}\left( t \right)dt}$; $s_{i,j}\left( \omega\right)=\frac{\int_{0}^{\infty} I_{i,j}\left( t \right)\sin\left( \omega t \right)dt}{\int_{0}^{\infty} I_{i,j}\left( t \right)dt}$ where *i*, *j* are the pixel coordinates in the FLIM image, ω=2π*f* the angular frequency and *f* the laser repetition rate (80 MHz in our data). Parameters *g* and *s* can be expressed in terms of *m* modulation and *φ* phase angle (Figure 1b), used to estimate apparent phase $\tau_{\varphi}=\frac{1}{\omega}\tan(\varphi)$ and modulation $\tau_{m}=\frac{1}{\omega}\sqrt{(\frac{1}{m^{2}}-1)}$ lifetimes. For single exponential decays (Figure 1b), these two lifetimes are equal and the phasor coordinates *g* and *s* lie on the universal semicircle with radius 0.5 going from point (1, 0) to point (0, 0), corresponding respectively to $\tau=0$ and $\tau=\infty$. On the contrary, the phasor coordinates of mixed A&B species lie inside the universal semicircle along a line connecting the two distinct lifetime phasors (A and B). The phasor plot of an *n*-component mixture will reside within a polygon with *n*-vertices located in the position of the phasor of each contributing species. Its coordinates are given by $G\left( \omega\right)=\sum_{n} f_{n}g_{n}\left( \omega\right)$ and $S\left( \omega\right)=\sum_{n} f_{n}s_{n}\left( \omega\right)$ where the relative contributions are normalized $\sum_{n} f_{n}=1$.

## Pseudo‑FLIM analysis for 2D and 3D melanin detection

In this section we describe the influence of temporal binning and maximum intensity peak position (0 ns, 1.33 ns) on the Pseudo-FLIM slope parameter.

In our time-domain lifetime measurements (and in most of the multiphoton FLIM human skin publications), the first-time channels before the rising edge of the fluorescence are usually found within an approximately ~1ns time window and correspond to noise photons (e.g. used to estimate the “offset” parameter in the FLIM bi-exponential analysis with SPCImage software). At 1 ns starts the rising edge of the fluorescence and the maximum intensity (peak position) is found at ~1.33 ns.

We studied the influence of the temporal binning (time / channel of 1.5 ns, 2 ns and 3 ns) on the Pseudo-FLIM slope parameter using simulated intensity decays for melanin (A&B mixed species from Figure 1a) and for a mixture of free and bound NADH (70% relative contribution of free NADH ($\tau_{1}$=0.4 ns) and 30% of bound NADH ($\tau_{2}$=2.5 ns)). The longer the integration time, the higher the 2PEF intensity of the first t-channel and consequently the higher the slope is. When calculating the ratio between the slopes of simulated “melanin” and "free/bound NADH" data, the higher ratio was obtained for 1.5 ns (3.77) and 2 ns (1.44) integration times. Although, this ratio is higher for 1.5 ns time window, the signal intensity of the first time-channel (integral of noise photons (1 ns time window), rising edge fluorescence photons and a few fluorescence photons within ~0.2 time window after the peak position) is lower compared to the 2 ns temporal binning and the slopes are also smaller.

We also studied the influence of the temporal binning (time / channel of 1.5 ns, 2 ns, 2.5 ns and 3 ns) on real 2PEF intensity decays: see the “melanin” and “other constituents” data in Figure 4b1. The results (Figure S1) show the same trend as for the simulated data. Figures S1a and b show the effect on the 2PEF intensity decays (first 3 temporally binned time-channels) of "melanin" and "other constituents". The decays are transformed in ln(2PEF Intensity) and a linear regression is performed to extract the slope of the decay (Figure S1c, d). The increase in slope parameter (Figure S1e, d) with the integration time is clearly evidenced for both conditions. As for the simulated data, the higher ratio between the slopes of “melanin” and "other constituents" (Figure S1c, d) was obtained for 1.5 ns (2.56) and 2 ns (1.42) integration times, but the signal intensity of the first time-channel is lower at 1.5 ns compared to 2 ns temporal binning and the slopes are also smaller.

Also, varying the maximum peak position from 1.33 ns to 0 ns (for a fixed 2 ns integration time) allows increasing the slope parameter value, but the “melanin” to "other constituents" slopes ratio is much smaller (idem for the simulated data).

Given these considerations, we chose to work with the 2 ns temporal binning and 1.33 ns 2PEF intensity peak position, but if these parameters are changed, one will have to adapt the slope threshold in order to discriminate melanin from the other constituents.

## *In vivo* 3D automatic skin layers segmentation and constituents quantification

Based on mathematical morphology and graph theory, an automatic epidermis / dermis 3D segmentation (Figure 2a) is computed followed by a 3D segmentation of *stratum corneum* (SC) and living epidermis (LED) sub-layers. The z-stack of 2PEF-FLIM (4 time channels, 2 ns time/channel and 1.33 ns maximum peak position) is further processed for Pseudo-FLIM melanin detection (Figure 2b). The 3D z-stack of melanin masks and automatic epidermis segmentation are jointly used for 3D melanin quantification (Figure 2c). The 3D reconstruction of melanin masks allows visualizing melanin distribution within the epidermis. We defined global 3D epidermal melanin density as the ratio between the numbers of melanin voxels to epidermal voxels. The 3D melanin density can also be estimated within the epidermal sub-layers (e.g. living epidermis LED or a fixed 10 µm layer above the DEJ, mainly corresponding to the basal layer).

MPSTS software also enables a more refined epidermis segmentation, by defining 10 or 12 thickness-normalized epidermal sub-layers that follow the 3D shape of the DEJ and SC. Melanin density can be computed in each sub-layer and a melanin z-epidermal distribution profile can be extracted.

Finally, the software extracts 3D morphological parameters (e.g. SC, LED and epidermis mean thickness, normalized DEJ area characterizing DEJ undulation in 3D) and 3D density parameters (e.g. melanin, elastin and fibrillar collagens fibers).

# Supplementary Figures

**Figure S1: Effect of temporal binning – increase in the integration time/channel – on Pseudo-FLIM slope parameter**. The “melanin” and “other constituents” FLIM 2PEF intensity decays were acquired *in vivo* on human forearm skin and correspond to the data of Figure 4 b1 (see the insert). (top) 2PEF intensity decays of a) melanin and b) other constituents for different integration times /channel (t-channel – temporally binned channel). (middle) Graphs c) and d) show the natural logarithm transformation and linear regression fitting to extract the slopes of the decays. The increase in the slopes with the integration time is clearly visualized for both c) melanin and d) other constituents’ data. (bottom) Changes in Pseudo-FLIM slope parameter with the integration time for e) melanin and c) other constituents. The ratio between the slopes of “melanin” and “other constituents” is given in the white rectangles in graph e).

**Figure S2: Additional FLIM bi-exponential fitting and Phasor data for melanin detection *in vivo* on human skin.** Multiphoton 2D 2PEF FLIM images acquired at different depths from the skin surface to the dermis, within *stratum corneum* (SC) *disjunctum*, *corneum compactum*, *granulosum* (SG), *spinosum* (SS), *basale* (SB) and superficial dermis were analyzed with the three methods. (top) FLIM bi-exponential fitting analysis images of the amplitude-averaged fluorescence lifetime τ_m_ and relative contribution a1[%] of the short τ_1_ fluorescence lifetime. (bottom) Enlarged view of the phasor plots (*s* versus *g* scatters) of the different skin layers from Figure 4, color coded using the same color scale as for τ_1_ and τ_2_ parameters as in Figure 4.


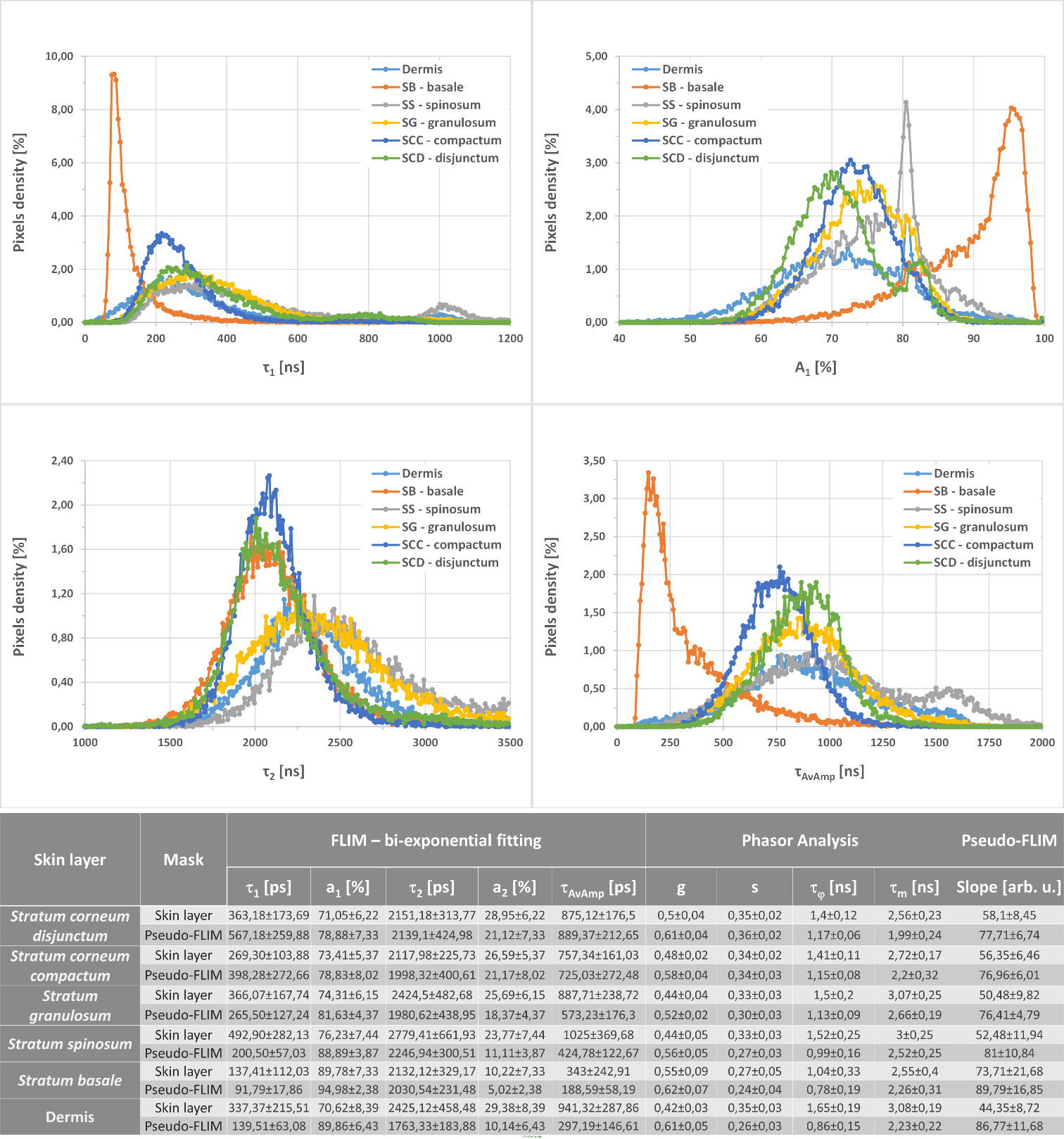


**Figure S3: Quantification of FLIM bi-exponential fitting, Phasor and Pseudo-FLIM images of *in vivo* human skin.** (top) Histograms of FLIM bi-exponential parameters and (bottom) mean with standard deviation of the mean for the images shown in Figure 4. The multiphoton 2D 2PEF FLIM images were acquired at different depths from the skin surface to the dermis, within stratum *corneum* (SC) *disjunctum*, *corneum compactum*, *granulosum* (SG), *spinosum* (SS), *basale* (SB) and superficial dermis, and were analyzed with the three methods. FLIM bi-exponential analysis parameters: τ_1_ –short and τ_2_ long-fluorescence lifetimes and a_1_[%], a_2_[%] their respective relative contributions; τ_AvAmp_ –amplitude weighted average lifetime. Phasor analysis parameters: *g*, *s*, τ_φ_ phase lifetime and τ_m_ modulation lifetime parameters. Pseudo-FLIM analysis parameter: slope.

**Figure S4: Comparison of *in vivo* human skin melanin masks obtained with FLIM bi-exponential fitting, Phasor and Pseudo-FLIM analyses.** Within each skin layer, the melanin masks were obtained upon application of different parameters depending thresholds indicated on the right side of the figure. For Pseudo-FLIM melanin quantification, these masks are further processed with an open area filter to remove the isolated regions of interest with an area smaller than the approximated 1µm² melanosomal area.

**Figure S5: Skin ventral forearm color measurements in the constitutive pigmentation study.** The data of L* - skin lightness, a* - skin red-green component, b* - skin blue–yellow component and ITA – individual typological angle calculated based on L* and b* parameters are expressed as boxplots with fences.

**Figure S6:** **Modulation of global 3D melanin density in different epidermal sub-layers with skin color ITA grade in constitutive pigmentation study.** The data are expressed as boxplots with fences. ED (global epidermis), SC - *stratum corneum*, SC Disj - *stratum corneum disjunctum*, SC Comp - *stratum corneum compactum*, LED – living epidermis, SG - *stratum granulosum*, SS – *stratum spinosum* and SB - *stratum basale*. The 3D melanin density in a specific epidermal sub-layer is expressed as % of the volume of that layer; the epidermal sub-layers have different volumes.

**Figure S7:** **Modulation of global 3D melanin density and z-epidermal distribution between ventral and dorsal forearm sides in photo-aging study.** Example of mosaic images extracted from a z-stack of raw 2PEF intensity images (cyan hot) of a) ventral and b) dorsal forearm side of an old volunteer. Corresponding Pseudo-FLIM melanin masks (white) of c) ventral and d) dorsal sides. e) Changes in 3D epidermal melanin density with aging and forearm side. The data are presented as boxplots with fences. f) Changes with aging and forearm side in the mean profile of 3D melanin z-epidermal distribution from DEJ – dermal-epidermal junction to SC - *stratum corneum*. The data are presented as mean with 95% confidence interval of the mean.

**Figure S8: Short-term retinoids effects on melanin density under occlusive application**. At the top left, quantification of melanin density obtained with multiphoton microscopy in the living epidermis expressed in % of skin volume for Control, Retinol (RO) and Retinoic acid (RA)-treated areas (a). The data are expressed as boxplots with fences. The boxes contain 50% of the data; the intervals between the lower limit of the box and the lower inner fence contain 25%, and vice versa for the other 25%. — indicates the median that divides the population in two groups with equal numbers of data points; ° the outliers and * the extreme data points. At the top right, standard photography of the dorsal side of the left forearm of a volunteer showing the RO, Control and RA-treated areas showing a clear whitening of the skin on RO-treated area and a more discreet whitening on the RA-treated area (b). At the bottom, in cyan hot LUT, Pseudo-FLIM melanin masks color coded by the 2PEF signal intensity within the basal layer of Control, RO and RA-treated areas (c) at D32. *Figure reprinted from J. Eur. Acad. Dermatol. Venereol., 29, Trancrède-Bohin et al., Non-invasive short-term assessment of retinoids effects on human skin in vivo using multiphoton microscopy, 673-681, Copyright (2015), with permission from John Wiley and Sons Ltd*.


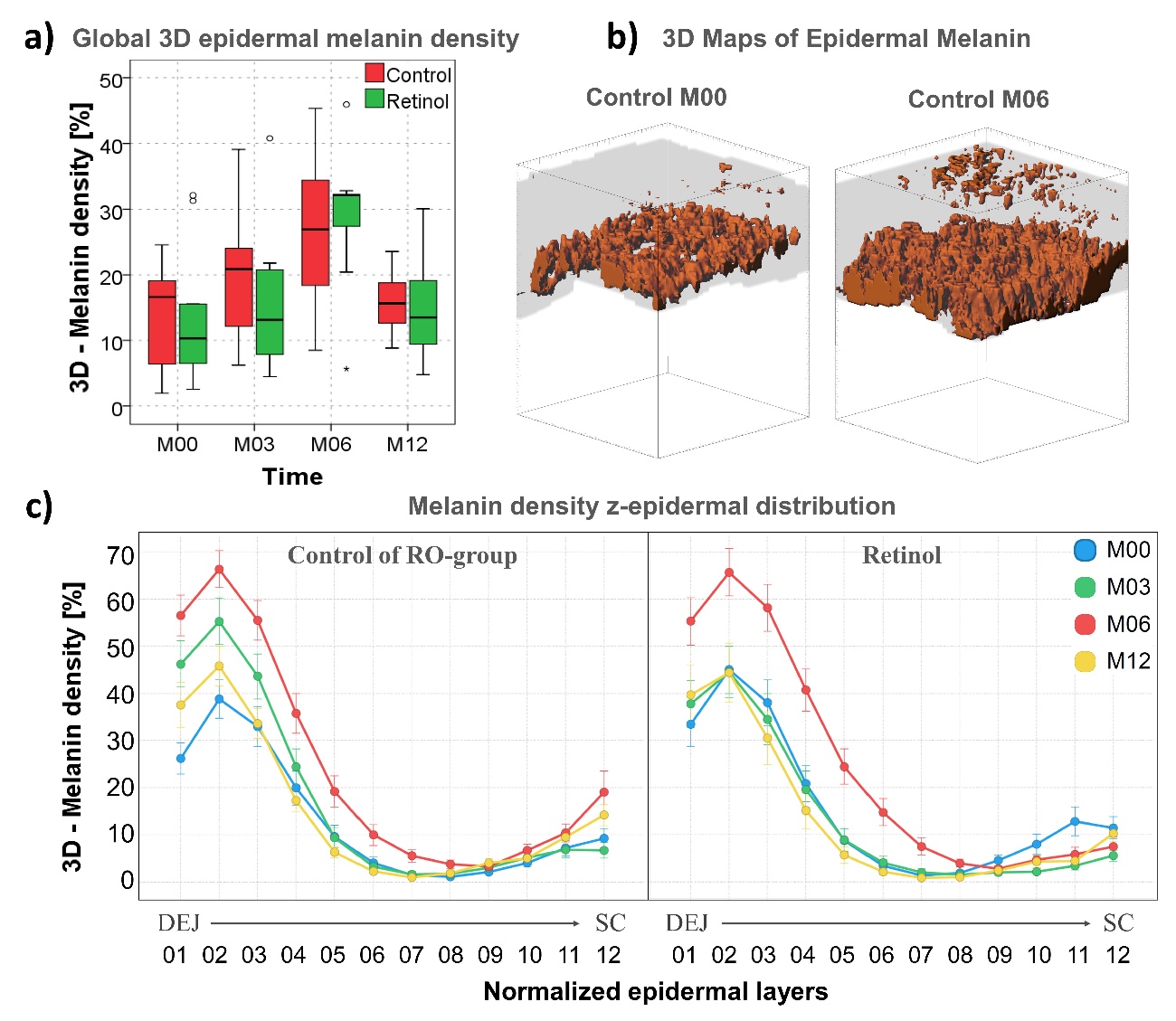


**Figure S9: Modulations over 1 year of melanin global density and z-epidermal distribution with seasonality and retinoids**. a) Change with time in the global 3D epidermal melanin density at months M00 (March), M03 (June), M06 (September), M12 (March +1year). The data are expressed as box plots with fences. b) 3D maps of melanin melanin of a representative control subject at M00 and M06. These 3D reconstructions were created with Imaris (Bitplane AG, Zürich, Switzerland) software; c) Melanin density z-epidermal distribution profiles (mean 3D melanin density estimated in 12 thickness-normalized epidermal layers from 1—DEJ level to 12—SC level). The z-profiles data are expressed as mean ± SEM. *Figure adapted from Skin Res. Technol., 26, Trancrède-Bohin et al., In vivo multiphoton imaging for non-invasive time course assessment of retinoids effects on human skin, 794-803, Copyright (2020), with permission from John Wiley and Sons Ltd*.

**Table S 1:** **Skin ventral forearm color and 3D melanin density measurements in the constitutive pigmentation study.** For each skin color ITA group, from I – very light to V – brown skin color, the min, max, mean, median, Q1 and Q3 first and third quartile, SD – standard deviation of the mean and the MAD – median absolute deviation values are given for each measurement. Colorimetric measurements: L* - skin lightness, a* - skin red-green component, b* - skin blue–yellow component and ITA – individual typological angle calculated based on L* and b* parameters. The 3D multiphoton melanin density was estimated using the Pseudo-FLIM approach in ED (global epidermis), SC - *stratum corneum*, SC Disj - *stratum corneum disjunctum*, SC Comp - *stratum corneum compactum*, LED – living epidermis, SG - *stratum granulosum*, SS – *stratum spinosum* and SB - *stratum basale*.
